# The Cerebrospinal Fluid Free-Glycans Hex_1_ and HexNAc_1_Hex_1_Neu5Ac_1_ as Potential Biomarkers of Alzheimer’s Disease

**DOI:** 10.3390/biom14050512

**Published:** 2024-04-24

**Authors:** Lynn Krüger, Karina Biskup, Carola G. Schipke, Bianca Kochnowsky, Luisa-Sophie Schneider, Oliver Peters, Véronique Blanchard

**Affiliations:** 1 Institute of Diagnostic Laboratory Medicine, Clinical Chemistry, Charité—Universitätsmedizin Berlin, Corporate Member of Freie Universität Berlin and Humboldt-Universität zu Berlin, Augustenburger Platz 1, 13353 Berlin, Germany; lynn.krueger@medicalschool-berlin.de (L.K.);; 2 Department of Human Medicine, Medical School Berlin, Rüdesheimer Str. 50, 14197 Berlin, Germany; 3 Department of Psychiatry and Psychotherapy, Charité—Universitätsmedizin Berlin, Corporate Member of Freie Universität Berlin and Humboldt-Universität zu Berlin, Hindenburgdamm 30, 12203 Berlin, Germany; schipke@predemtecdx.com (C.G.S.); bianca.kochnowsky@charite.de (B.K.); luisa-sophie.schneider@charite.de (L.-S.S.); oliver.peters@charite.de (O.P.)

**Keywords:** free glycans, N-glycans, O-glycans, biomarker, Alzheimer’s disease

## Abstract

Alzheimer’s disease (AD) is the most common neurodegenerative disorder, affecting a growing number of elderly people. In order to improve the early and differential diagnosis of AD, better biomarkers are needed. Glycosylation is a protein post-translational modification that is modulated in the course of many diseases, including neurodegeneration. Aiming to improve AD diagnosis and differential diagnosis through glycan analytics methods, we report the glycoprotein glycome of cerebrospinal fluid (CSF) isolated from a total study cohort of 262 subjects. The study cohort consisted of patients with AD, healthy controls and patients suffering from other types of dementia. CSF free-glycans were also isolated and analyzed in this study, and the results reported for the first time the presence of 19 free glycans in this body fluid. The free-glycans consisted of complete or truncated N-/O-glycans as well as free monosaccharides. The free-glycans Hex_1_ and HexNAc_1_Hex_1_Neu5Ac_1_ were able to discriminate AD from controls and from patients suffering from other types of dementia. Regarding CSF N-glycosylation, high proportions of high-mannose, biantennary bisecting core-fucosylated N-glycans were found, whereby only about 20% of the N-glycans were sialylated. O-Glycans and free-glycan fragments were less sialylated in AD patients than in controls. To conclude, this comprehensive study revealed for the first time the biomarker potential of free glycans for the differential diagnosis of AD.

## 1. Introduction

Alzheimer’s disease (AD), characterized by slow cognitive decline, is the most common neurodegenerative disorder in elderly people and represents about 70% of diagnosed cases of dementia [1]. In 2021, it was estimated that about 55 million people were affected by dementia worldwide and predictions state 139 million cases by 2050, whereby the greatest increase is expected in developing and semi-developed countries [2,3]. In AD demented individuals, neurodegeneration results from the formation of amyloid plaques and from the aggregation of the tau protein. The amyloid β-peptide (Aβ) is a 4 kDa peptide that is cleaved by the secretase complex (β- and γ-secretase) from the amyloid precursor protein (APP) present in neuronal membranes [4]. After cleavage, the amyloid β-peptide switches from a random coil or α-helix conformation to a β-hairpin, which facilitates its polymerization and accumulation into plaques [5]. In addition, abnormal phosphorylation of the intracellular microtubule-associated protein tau plays an important role in the neurobiological processes underlying AD. The O-GlcNAcylation of the tau protein was found to regulate the phosphorylation of tau in AD patients [6,7,8]. The downregulation of tau O-GlcNAcylation results in hyperphosphorylation, which renders the tau protein dysfunctional [9]. The ability to promote assembly and to stabilize microtubules is not only lost but hyperphosphorylated tau sequesters normal tau and other neuronal microtubule-associated proteins, leading to the formation of neurofibrillary tangles [10]. The disruption of the microtubule network can affect axonal transport, causing retrograde degeneration and further dementia [11]. 

Although age, sex or other exogenous factors, such as traumatic brain injury or a persons’ lifestyle, are the main causes of Alzheimer’s disease, there is also a rare genetic origin; about 1% of dominantly inherited familiar AD is caused by mutations of the genes coding for the amyloid precursor protein, presenilin-1 and presenilin-2 [12,13,14,15,16]. Moreover, the isoform ε4 of the cholesterol transporter apolipoprotein E is considered a high genetic risk factor for AD [17]. Knowing the evolution of dynamic biomarkers, it is estimated that neuronal degeneration also starts occurring in the brain as early as 10–30 years before the first symptoms are observed in the most common spontaneous cases of AD [18,19]. When the brain is not able to compensate for the neuronal degeneration, the first signs of mild cognitive impairment (MCI) will appear [20]. Patients with MCI suffer from noticeable changes in their thinking abilities but are still able to do their daily activities, whereas the daily life of AD patients is impaired due to more severe cognitive losses [20].

The diagnosis of AD is based on history and physical examination, psychological testing, brain-imaging and CSF biomarkers to exclude other brain diseases, but the definitive diagnosis of AD can only be carried out post-mortem by a neuropathological examination [21]. Generally, there are no Alzheimer’s-related physical changes. However, in rare cases, affected individuals showed signs of progressive apraxia, aphasia or visual impairment [22,23,24,25,26,27,28,29]. Psychological testing is performed by clinicians through questionnaires such as the Mini-Mental State Examination (MMSE). At a molecular level, fibrillar Aβ can be visualized with positron emission tomography using ^11^C-labeled Pittsburgh compound B (PiB) or ^18^F-based tracers, such as NeuraCeq^TM^ or Amyvid^®^ [30,31,32,33]. These compounds bind to insoluble fibrillary amyloid plaques. However, tracer binding is observed in patients suffering from dementia with Lewy bodies as well as in healthy elderly; e.g., 30–40% at the age of 80 years show high PiB binding [34]. Computed tomography scanning and magnetic resonance imaging serve as further imaging techniques. CSF is in direct contact with the extracellular space of the brain and reflects to a certain extent its biochemical status. The collection of CSF is invasive, but it remains the best body fluid to perform a biomarker-based analysis of the type of brain disorders, although serum-based biomarkers have been developed in recent years but are yet to be implemented in the routine diagnostics [19]. In order to support the diagnosis of AD, the total tau protein (t-tau), phosphorylated tau (p-tau), β-amyloid proteins Aβ_1–42_ (Aβ)/Aβ_1–40_ and the ratio Aβ_1–42_/Aβ_1–40_ are the markers that are quantified routinely in CSF, but they are not sensitive and specific enough to be used exclusively; this is why they are used in combination with imaging techniques [19,35]. High t-tau and p-tau correlate with a fast progression of cognitive decline, corresponding to a transition from MCI to AD [36,37]. The Aβ-peptides, which are secreted during cellular metabolism as normal components of the CSF [38,39], are downregulated by 50% in AD patients [40]. Multiparameter assays combining the measurements of CSF t-tau, p-tau and Aβ-peptides have recently shown better diagnostic performances (sensitivity and specificity of 80–90%) than when the markers were analyzed alone [41,42]. However, it remains difficult to distinguish AD from other forms of dementia, such as Lewy body dementia and vascular dementia [43]. At a plasma level, biomarker discovery has so far been limited to pilot studies. For example, measurements of plasma Aβ were contradictory, possibly due to its hydrophobicity, which makes it prone to plasma protein binding, resulting in inaccurate measurements [44].

Glycosylation is a protein post-translational modification that is essential for cell–cell recognition processes, cell differentiation and development, immune response, secretion and protein processing and folding [45,46,47]. Glycans are modulated in many inborn and acquired diseases, such as congenital disorders of glycosylation, infections, immune reactions or even in the development or detection of cancer [48,49,50,51,52]. A correlation was already established between glycome profiles of the brain and biochemical modifications of the corresponding glycosyltransferases in the context of neurological diseases such as AD [53,54,55,56,57]. A decrease in sialyltransferase levels and activity were reported in patients with AD [56,57,58]. In addition, increased expression of *N*-acetylglucosaminyltransferase III (GnT-III), the enzyme responsible for bisection [54], correlated with increased levels of bisecting GlcNAc in CSF [59].

Due to the growing prevalence of Alzheimer’s disease and the rising interest in reliable biomarkers for the early diagnosis of the disease, we evaluated the glycoprotein glycome as a possible source of biomarkers. In recent years, several groups have established analytical workflows designed to analyze N-glycome in CSF [60,61,62]. Interestingly, Fogli et al. and Goyallon et al. reported a permethylated glycan structure at *m*/*z* 1601. Goyallon and coworkers [60] showed using ion trap tandem mass spectrometry (MS) that this signal corresponded to Neu5Ac_3_Hex_1_HexNAc_1_, suggesting the presence of free-glycans in the CSF samples. In other words, non-covalently attached glycans derived from glycoproteins are present in CSF. The occurrence of free-glycans has been well described for serum [63,64], urine [65,66,67], and seminal plasma [68], but not for CSF so far. This is why we developed a multi-step workflow to examine the entire glycoprotein N-, O- and free-glycome from CSF and plasma using a large cohort composed of 262 patients (Alzheimer’s, other types of dementia and healthy controls). Our aims were, first, to study the free-glycans in detail and then to propose sets of glycans as biomarkers for AD that were modulated differently in controls or in the other types of dementia.

## 2. Materials and Methods

### 2.1. Materials

Trypsin, sodium dihydrogen phosphate monohydrate, di-sodium hydrogen phosphate dihydrate, 1,4-dithioerythritol (DTE), 2-iodoacetamide (IAA), sodium borohydride, Dowex 50WX8 ion exchange resins, sodium hydroxide (NaOH), iodomethane, sDHB (9:1 mixture of 2,5-dihydroxybenzoic acid and 2-hydroxy-5-methoxy benzoic acid) and dextran were purchased from Merck (Taufkirchen, Germany). Acetonitrile (ACN) and anhydrous dimethyl sulfoxide (DMSO) were purchased from VWR International GmbH (Dresden, Germany). Trifluoroacetic acid (TFA) and acetic acid were purchased from Thermo Fisher Scientific (Schwerte, Germany). Peptide N-glycosidase F (PNGase F) was purchased from N-Zyme Scientifics (Doylestown, PA, USA). Methanol (MeOH) was purchased from Carl Roth GmbH + Co. KG (Karlsruhe, Germany). Milli-Q grade water was produced using a MilliQ Plus water purification system (Merck KGaA, Darmstadt, Germany). All the reagents were obtained in p.a. quality.

### 2.2. Patient Population

The patient populations used in this study consisted of 89 patients with AD, 86 disease control patients (patients diagnosed with frontotemporal dementia, dementia with Lewy bodies or Parkinson’s dementia) and 87 healthy controls (fully characterized healthy controls with no cognitive concerns). The Ethics Committee of the Charité—Universitätsmedizin Berlin, Germany (EA4/175/16 and EA4/066/17) approved the use of the samples.

### 2.3. Sample Collection and Characterization

CSF was collected and analyzed according to a standardized protocol [69]. Briefly, 12 mL of CSF was collected into polypropylene tubes. Immediately after collection, the tubes were gently shaken and centrifuged (room temperature, 2000× *g*, 10 min), aliquoted (500 µL), frozen within 30 min and stored at −80 °C. To quantify Aβ peptides in CSF, the Lumipulse^®^ G β -Amyloid 1–42 and Lumipulse^®^ G β -Amyloid 1–40 assays (Fujirebio Germany GmbH, Hannover, Germany) were used. For total (t-)tau and phosphorylated (p-)tau quantification, the Lumipulse^®^ G Total Tau and Lumipulse^®^ G p-Tau 181 assays (Fujirebio Germany GmbH, Hannover, Germany) were used, respectively. All the measurements were carried out fully automatically on the LUMIPULSE^®^ G600II instrument. Under these conditions, the following CSF biomarker values were rated as indicative of AD: Aβ (1–42) < 680 pg/mL, Aβ (1–42)/Aβ (1–40) ratio < 0.055, t-tau > 400 pg/mL and p-tau > 62 pg/mL. Patient diagnosis was performed using a combination of MMSE scoring, brain imaging and CSF parameters (Aβ40, Aβ42, Aβ-ratio, p-tau, t-tau). The patients’ data is summarized in Table 1.

### 2.4. Isolation and Purification of Free-Glycans

Aliquots of 100 µL CSF were used for glycome analysis. Samples were enzymatically digested with trypsin (15 µg) immediately after thawing. Overnight incubations were performed at 37 °C for at least 16 h. The following day, trypsin was heat-inactivated for 5 min at 95 °C and samples were acidified with trifluoroacetic acid (TFA) to a final concentration of 0.1%. Free-glycans were isolated from CSF using reversed-phase C4 solid-phase extraction cartridges (Macherey-Nagel, Düren, Germany). Cartridges were pre-equilibrated according to the manufacturer’s instructions. After equilibration, acidified samples were added, and cartridges were washed three times with 0.1% TFA. The sample flow-through, containing free-glycans, was collected and subsequently desalted on Supelclean ENVI-Carb solid-phase extraction cartridges (Sigma-Aldrich Chemie, Hamburg/Taufkirchen, Germany). Afterwards, free-glycan samples were gradually eluted with 25% and 50% aqueous acetonitrile (ACN) containing 0.1% TFA and dried under reduced atmospheric pressure by using a centrifugal vacuum concentrator [70].

### 2.5. N-Glycan Release and Purification

Glycopeptides bound to the C4 cartridges were gradually eluted using 25%, 50% and 80% aqueous ACN containing 0.1% TFA and subsequently dried under reduced atmospheric pressure as well. Dried samples were dissolved in 200 mM phosphate buffer (pH 6.5), reduced with 200 mM DTE at 60 °C for 45 min and alkylated with 200 mM IAA at room temperature for 1 h in the dark. The reaction was terminated using 200 mM DTE. Samples were diluted with Milli-Q water and 200 mM phosphate buffer. PNGase F (1 μg/sample) was added to release N-glycans and the reaction mixture was incubated overnight at 37 °C. N-Glycans were isolated from peptides, desalted, and dried as previously described in 2.4 for free-glycans.

### 2.6. O-Glycan Release and Purification

De-N-glycosylated peptides eluted from the C4 cartridges during N-glycan purification were dried in a vacuum concentrator. Then, samples were resuspended with 1 M sodium borohydride in 0.1 M NaOH. Chemical release of O-glycans was allowed to proceed overnight at 45 °C. The next day, samples were placed on ice and 10% aqueous acetic acid was added until gas development stopped (pH-value ≤ 4). O-Glycans were isolated using C18 solid-phase extraction cartridges (Sigma-Aldrich Chemie, Hamburg/Taufkirchen, Germany) filled with Dowex 50WX8 ion exchange resins in H^+^ form. Prior to that, C18 cartridges were pre-equilibrated with 100% MeOH. Afterwards, Dowex was added and the cartridges were washed three times with MeOH and 10% aqueous acetic acid. After adding the samples, the resulting flow-through and three washing fractions, containing O-glycans, were collected. O-Glycan samples were dried under reduced atmospheric pressure using a centrifugal vacuum concentrator. Salts were finally evaporated by adding 500 μL MeOH and then drying the sample in a vacuum concentrator. This step was repeated five times.

### 2.7. Permethylation

Permethylation was carried out according to standard protocols [71,72]. Briefly, glycan samples were permethylated using freshly prepared NaOH/DMSO suspension (~0.2 g NaOH per mL of DMSO) and iodomethane. Finally, the reaction was stopped by addition of chloroform. The organic phase was washed with water several times until the pH of the water phase became neutral. The inorganic phase was evaporated under a reduced atmosphere. Finally, samples were dissolved in 75% aqueous ACN for MALDI-TOF measurements.

### 2.8. Mass Spectrometry

Each glycan sample (1 μL) was mixed on target with 1 μL matrix (5 μg/μL sDHB in 10% aqueous ACN). The mixture was allowed to dry at room temperature. MALDI-TOF-MS spectra were acquired in positive reflector mode on an Ultraflex III mass spectrometer (Bruker Daltonics, Bremen, Germany), equipped with a Smartbeam laser (337 nm) and a LIFT-MS/MS facility. Calibration was performed using a glucose ladder. In total, 5000 laser shots were accumulated for each mass spectrum. Spectra for free-glycan and O-glycan analyses were recorded in the mass range of 0–3000 Da, whereas a mass range of 1000–5000 Da was used for N-glycans. Collision-induced dissociation was performed using a collision energy of 29 kV. LIFT spectra consisted of at least 2500 laser shots. FlexAnalysis version 3.4 (Bruker Daltonics, Bremen, Germany) was used for baseline correction and peak picking. Glycan structures were annotated using GlycoPeakfinder [73] and assigned glycan structures were generated with the GlycoWorkbench software version 2.1 [74,75]. Relative areas were determined for all glycan structures in all the samples.

### 2.9. Statistical Analysis

Statistical analysis was performed using SPSS version 27 (IBM, Armonk, NY, USA). Before a comprehensive statistical analysis was conducted, a two-way analysis of variance was carried out to assess whether the datasets were gender-dependent (Supplementary Material Tables S1–S3). This was the case for N-glycans and O-glycans but not for free-glycans. Then, a one-sample Shapiro–Wilk test was carried out to estimate the distribution normality. Due to a skewed distribution between the groups, non-parametric tests were used for the following analysis. A Mann–Whitney U test was used when comparing two groups and a Kruskal–Wallis one-way analysis of variance was used when comparing the three disease groups. *p*-values ≤ 0.05 were considered as statistically significant and *p*-values ≤ 0.01 as highly significant. Receiver operating characteristic curves were built with a 95% confidence interval. The correlations between the glycans, patient- and CSF-parameters were assessed using the Spearman’s rank correlation coefficient.

## 3. Results

Aiming at defining a set of glycan biomarkers specific to Alzheimer’s disease, we present here a comprehensive study of the free-, N- and O-glycans in the CSF and plasma. For this purpose, a total of 262 CSF and plasma samples from the same patients were analyzed by MALDI-TOF-MS. 

### 3.1. Human CSF Contains Free-, N- and O-Glycans

**Figure 1:**
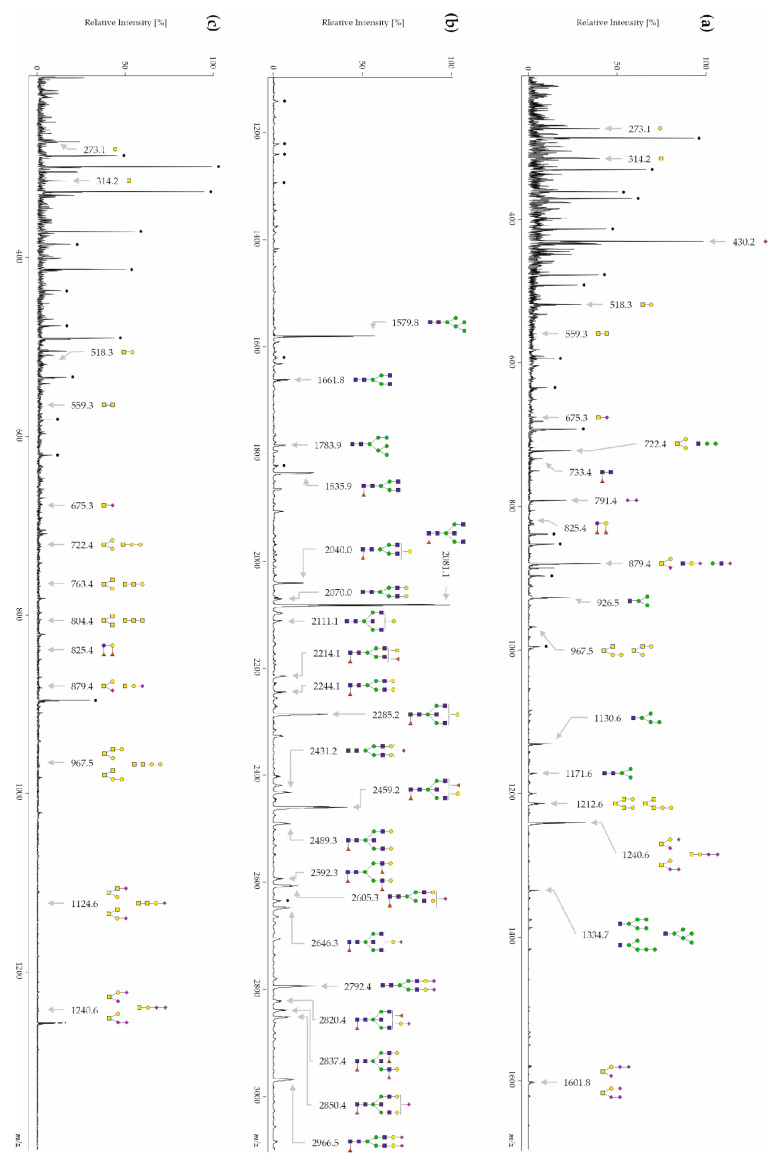
Representative MALDI-TOF-MS spectra of permethylated (**a**) free-glycans, (**b**) N-glycans, and (**c**) O-glycans isolated from healthy controls. Measurements were performed in the positive-ion mode. All the ions are present in their sodiated form [M + Na]^+^. The monosaccharides are depicted as follows: Man, green circle; Gal, yellow circle; GlcNAc, blue square; GalNAc, yellow square; Fuc, pink triangle; Neu5Ac, pink diamond; star polygon, non-identified.

A tryptic digestion of CSF samples was performed prior to the separation of free-glycans from glycoproteins via C4 cartridges in order to increase protein recovery. In our hands, protein recovery was extremely low when native CSF glycoproteins were applied directly to C4 cartridges or if a protein denaturation was performed prior to the C4 purification. Free-glycans, which did not bind to C4 cartridges, were collected in the flow-through and desalted using graphite cartridges. Subsequently, they were permethylated and then measured by MALDI-TOF-MS in the positive ionization mode. Measurements in the negative reflector mode did not lead to any signals. An exemplary MALDI-TOF mass spectrum is shown in Figure 1a. The results indicate that a total 19 free-glycans were detected in the mass range of *m*/*z* 0–3000 Da: two signals correspond to the free sialic acids Neu5Ac_1_ (*m*/*z* 430.1) and Neu5Ac_2_ (*m*/*z* 791.4), five signals correspond to N-glycans with an intact reducing end or with a truncated reducing end (Fuc_1_HexNAc_2_—*m*/*z* 733.4, Hex_3_HexNAc_1_—*m*/*z* 926.5, Hex_4_HexNAc_1_ *m*/*z* 1130.6, Hex_3_HexNAc_2_ *m*/*z* 1171.6 and Hex_5_HexNAc_1_—*m*/*z* 1334.7) and five signals coincide with O-glycans (Fuc_2_Hex_2_—*m*/*z* 825.4, Hex_2_HexNAc_2_—*m*/*z* 967.5, Hex_2_HexNAc_3_—*m*/*z* 1212.6, Neu5Ac_2_Hex_1_HexNAc_1_—*m*/*z* 1240.6 and Neu5Ac_3_Hex_1_HexNAc_1_—*m*/*z* 1601.8). The other seven signals (Hex_1_—*m*/*z* 273.1, HexNAc_1_—*m*/*z* 314.2, Hex_1_HexNAc_1_—*m*/*z* 518.3, HexNAc_2_—*m*/*z* 559.3, Neu5Ac_1_HexNAc_1_—*m*/*z* 675.3, Hex_2_HexNAc_1_—*m*/*z* 722.4 and Neu5Ac_1_Hex_1_HexNAc_1_—*m*/*z* 879.4) were assigned to glycans but could not be precisely assigned to N- or O-glycans because their fragmentation patterns suggested several underlying structures and/or the obtained fragments could correspond to both N- and O-glycan structures (Supplementary Material Figure S1).

CSF glycopeptides, eluted from the C4 cartridges, were reduced, alkylated and digested with PNGase F to release all the N-glycans. After C4 and graphite purification, N-glycan samples were subsequently permethylated and measured by MALDI-TOF-MS in positive reflector mode. An exemplary MALDI-TOF mass spectrum is shown in Figure 1b. A total of 64 N-glycan signals were detected in the mass range of *m*/*z* 1000–3000 (Supplementary Table S4). The two N-glycans detected at *m*/*z* 1579.8 and 1783.9 correspond to the high-mannose structures Hex_5_HexNAc_2_ and Hex_6_HexNAc_2_, respectively. Eleven MS signals correspond to complex-type biantennary N-glycans and are partially fucosylated and/or sialylated: *m*/*z* 1661.8 (Hex_3_HexNAc_4_), *m*/*z* 1835.9 (Fuc_1_Hex_3_HexNAc_4_), *m*/*z* 2040.0 (Fuc_1_Hex_4_HexNAc_4_), *m*/*z* 2070.0 (Hex_5_HexNAc_4_), *m*/*z* 2214.1 (Fuc_2_Hex_4_HexNAc_4_), *m*/*z* 2244.1 (Fuc_1_Hex_5_HexNAc_4_), *m*/*z* 2431.2 (Neu5Ac_1_Hex_5_HexNAc_4_), *m*/*z* 2592.3 (Fuc_3_Hex_5_HexNAc_4_), *m*/*z* 2605.3 (Neu5Ac_1_Fuc_1_Hex_5_HexNAc_4_), *m*/*z* 2792.4 (Neu5Ac_2_Hex_5_HexNAc_4_) and *m*/*z* 2966.5 (Neu5Ac_2_Fuc_1_Hex_5_HexNAc_4_). Based on previous studies of CSF N-glycans [76,77,78], the following MS signals were assigned to bisecting N-glycan structures: *m*/*z* 2081.1 (Fuc_1_Hex_3_HexNAc_5_), *m*/*z* 2111.1 (Hex_4_HexNAc_5_), *m*/*z* 2285.2 (Fuc_1_Hex_4_HexNAc_5_), *m*/*z* 2459.2 (Fuc_2_Hex_4_HexNAc_5_), *m*/*z* 2489.3 (Fuc_1_Hex_5_HexNAc_5_), *m*/*z* 2646.3 (Neu5Ac_1_Fuc_1_Hex_4_HexNAc_5_), *m*/*z* 2820.4 (Neu5Ac_1_Fuc_2_Hex_4_HexNAc_5_), *m*/*z* 2837.4 (Fuc_3_Hex_5_HexNAc_5_) und *m*/*z* 2850.4 (Neu5Ac_1_Fuc_1_Hex_5_HexNAc_5_). The above-mentioned N-glycan signals were analyzed by MALDI-TOF/TOF-MS (Supplementary Material Figure S2). After elution from the C4 cartridges, O-glycans were cleaved chemically from the de-N-glycosylated peptides using sodium borohydride. C18 cartridges filled with Dowex ion-exchange resins were used for the separation of O-glycans from de-O-glycosylated peptides. All the O-glycan samples were subsequently permethylated and measured by MALDI-TOF-MS in positive reflector mode. An exemplary MALDI-TOF mass spectrum is shown in Figure 1c. In this study, 13 O-glycan structures were detected in the mass range of *m*/*z* 0–3000, but MS signals were too low to generate MS/MS spectra. The detected O-glycans are composed of up to four monosaccharides and are partly sialylated. The monosaccharides Hex_1_ and HexNAc_1_ were detected at *m*/*z* 273.1 and 314.2, respectively. The signals at *m*/*z* 518.3, 559.3 and 675.3 correspond to the disaccharides Hex_1_HexNAc_1_, HexNAc_2_ and Neu5Ac_1_HexNAc_1_, respectively. The identified trisaccharides were Hex_2_HexNAc_1_ (*m*/*z* 722.4), Hex_1_HexNAc_2_ (*m*/*z* 763.4), HexNAc_3_ (*m*/*z* 804.4) and Neu5Ac_1_Hex_1_HexNAc_1_ (*m*/*z* 879.4). The tetrasaccharides consisted of Fuc_2_Hex_2_ (*m*/*z* 825.4), Hex_2_HexNAc_2_ (*m*/*z* 967.5), Neu5Ac_1_Hex_1_HexNAc_2_ (*m*/*z* 1124.6) and Neu5Ac_2_Hex_1_HexNAc_1_ (*m*/*z* 1240.6).

### 3.2. Inter-Day Reproducibility of Free-, N- and O-Glycans

Before applying the protocol to the cohort of patients with AD, the inter-day reproducibility was verified by analyzing the same CSF sample in triplicate on three consecutive days. The results are presented in Supplementary Material. The mean coefficients of variation for free-glycans were between 2.6 and 6.5 (Figure S3a), for N-glycans between 3.5 and 7.4 (Figure S3b) and for O-glycans between 3.3 and 7.7 (Figure S3c). All the values indicate the reproducibility of the applied method.

### 3.3. The Glycan Profiles of Patients with AD Differ Significantly from Healthy Controls and Other Types of Dementia

The patient cohorts consisting of 89 patients with AD, 86 patients suffering either from other types of dementia and 87 healthy controls were analyzed using the previously-mentioned reproducible workflow. 

#### 3.3.1. CSF Free-Glycans

**Figure 2:**
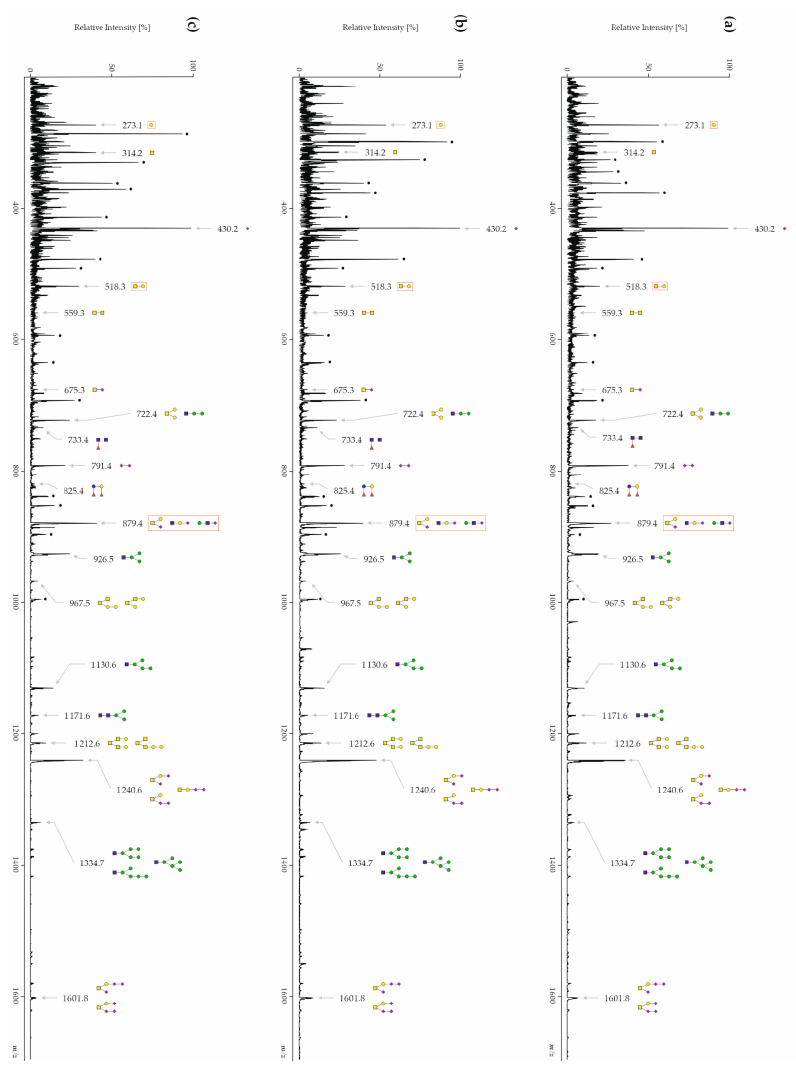
Exemplary MALDI-TOF-MS spectra of permethylated free-glycans from (**a**) a patient with AD (**b**) a disease control patient, and (**c**) a healthy control. Measurements were performed in the positive-ion mode. All the ions are present in their sodiated form [M + Na]^+^. The monosaccharides are depicted as follows: Man, green circle; Gal, yellow circle; GlcNAc, blue square; GalNAc, yellow square; Fuc, pink triangle; Neu5Ac, pink diamond; star polygon, non-identified. Free-glycans that are differentially expressed between the patient groups are encircled in red.

Relative intensities were determined for each glycan signal, and mean values and standard deviations are presented in Table 2. The glycan profiles of each patient group were compared between each other using the Kruskal–Wallis one-way analysis of variance as well as the Mann–Whitney U test. An upregulation of the free-glycan Hex_1_ (*m*/*z* 273.1) as well as a downregulation of Hex_1_HexNAc_1_ (*m*/*z* 518.3) and Neu5Ac_1_Hex_1_HexNAc_1_ (*m*/*z* 879.4) was observed in AD patients (Figure 2a) when compared with disease control patients (Figure 2b) and with healthy controls (Figure 2c). Patients with AD and disease control patients had significant statistical differences in 13 structures, whereas patients with AD and healthy controls had significant statistical differences in 14 glycan structures (Table 2). Interestingly, disease control patients and healthy controls also had statistical differences in six glycan structures (Table 2) and four of them could discriminate patients from each group: HexNAc_2_ (*m*/*z* 559.3), Neu5Ac_1_HexNAc_1_ (*m*/*z* 675.3), Hex_2_HexNAc_1_ (*m*/*z* 722.4) and Neu5Ac_1_Hex_1_HexNAc_1_ (*m*/*z* 879.4). 

ROC curves were built for the free-glycans that were of statistical significance, and the results are summarized in Supplementary Material Table S5. Interestingly, for both Hex_1_ and HexNAc_1_Hex_1_Neu5Ac_1_, it was possible to discriminate AD patients from healthy controls (area under the curve (AUC) 0.85 and 0.21) and AD patients from disease control patients (AUC 0.76 and 0.30). In addition, Hex_1_ and HexNAc_1_Hex_1_Neu5Ac_1_ correlated with the following CSF parameters: Aβ40, Aβ42, p-tau and t-tau (Supplementary Material Table S6). As AD patients and healthy controls had a mean age difference of about 10 years, we investigated any possible age correlation of the glycan biomarkers and verified the absence of such correlation (Supplementary Material Table S6).

#### 3.3.2. CSF N-Glycans

One-way ANOVA test showed gender-related statistical changes in the CSF N-glycome of the present cohorts (Supplementary Material Table S2). Therefore, the data was split according to gender prior to statistical analysis (Supplementary Material Table S7). A total of 18 N-glycans showed statistical differences between the three study cohorts (Table 3). The high-mannose N-glycans Hex_5_HexNAc_2_ (*m*/*z* 1579.8) and Hex_6_HexNAc_2_ (*m*/*z* 1783.9) were able to discriminate patients with AD and patients suffering from other types of dementia from healthy controls in male patients. In addition, complex-type biantennary N-glycans were also of statistical relevance. Interestingly, although many differences were gender-specific, the bifucosylated bisected biantennary N-glycan at *m*/*z* 2459.2 could statistically differentiate the three study cohorts from each other for both genders. In addition, the two sialylated N-glycans Neu5Ac_1_Fuc_1_Hex_5_HexNAc_4_ (at *m*/*z* 2605.3) and Neu5Ac_1_Fuc_1_Hex_4_HexNAc_5_ (at *m*/*z* 2646.3) could discriminate patients with AD from controls and patients with other forms of dementia for both genders.

#### 3.3.3. CSF O-Glycans

O-Glycans were detected as traces (Figure 1c), while non-carbohydrate contaminants were abundantly ionized. Relative areas of O-glycans from the three cohorts are shown in Supplementary Material Table S8. AD patients had significant statistical differences in eight O-glycan structures from disease control patients and in 11 O-glycan structures from healthy controls. Interestingly, disease control patients and healthy controls had significant statistical differences in all the structures at a significance level of *p* ≤ 0.01, and at a lower significance (*p* ≤ 0.05), at *m*/*z* 273.1 (female) and at *m*/*z* 1124.6 (male, *p* ≤ 0.05).

### 3.4. The Sialic Acid Content Is Significantly Decreased in Free-Glycan Fragments and O-Glycans from Patients with AD

**Figure 3:**
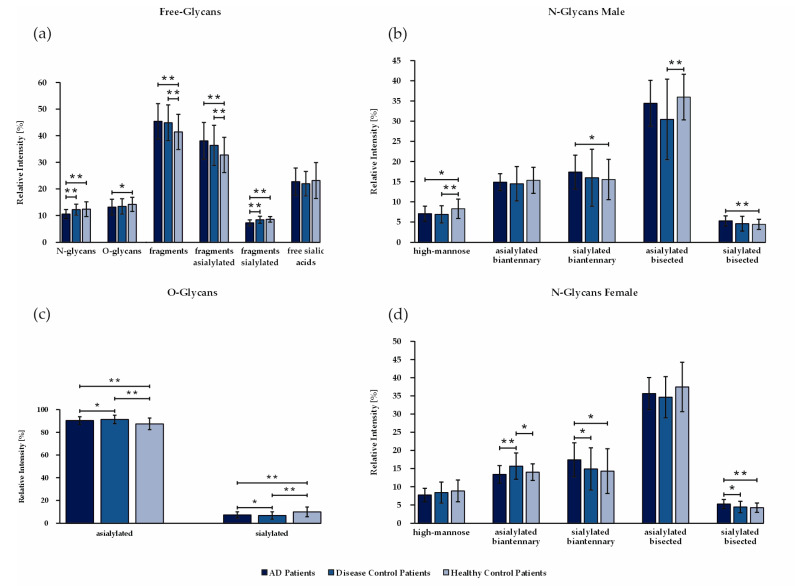
Bar charts showing the mean relative areas in (**a**) free-glycans, (**b**) N-glycans (male), (**c**) O-glycans and (**d**) N-glycans (female) for patients with AD, disease control patients and healthy controls. Glycans were grouped into glycosylation features, as detailed in Supplementary Material Table S9. * *p* < 0.5, ** *p* < 0.01.

Free- and N-glycans were combined according to their features (glycan types, sialylation, bisection, etc.) and their corresponding relative areas were summed up in order to identify the glycome features that were specific to only patients with AD (Figure 3a). The free-glycans were subgrouped into N-glycans (*m*/*z* 733.4, 926.5, 1130.6, 1171.6, 1334.7), O-glycans (*m*/*z* 825.4, 967.5, 1212.6, 1240.6, 1601.8), N- or O-glycan fragments (*m*/*z* 273.1, 314.2, 518.3, 559.3, 675.3, 722.4, 879.4) and free sialic acids (*m*/*z* 430.2, 791.4). Patients with AD, as well as patients with other types of dementia, had significantly less intact N- and O-glycans and more asialylated fragments than healthy controls. Similarly, N-glycan structures were combined into five main groups: high mannose structures (1579.8, 1783.9), biantennary asialylated (1661.8, 1835.9, 2040.0, 2070.0, 2214.1, 2244.1, 2592.3), biantennary sialylated (2431.2, 2605.3, 2792.4, 2966.5), asialylated bisecting structures (2081.1, 2111.1, 2285.2, 2459.2, 2489.3, 2837.4) and sialylated bisecting structures (2646.3, 2820.4, 2850.4). Sialylated biantennary and bisecting biantennary N-glycans were higher in patients with AD than in healthy controls both for the male and female cohorts (Figure 3b,d). As for free-glycan fragments, O-glycan sialylation was decreased in patients with AD compared to disease control patients and healthy controls (Figure 3c).

## 4. Discussion

This study reports for the first time a comprehensive analysis of free-, N- and O-glycans from CSF using a large cohort of patients with AD. This cohort was not only compared with healthy controls but also with patients with other forms of dementia. In this study, the serum N- and O-glycome from the same patients was also analyzed, but the analysis did not show statistically significant differences amongst the different patient groups and healthy controls. The study results demonstrated the presence of 19 free-glycans in CSF, in addition to 64 N- and 13 O-glycans. This number of N-glycans is in accordance with previous reports [59,78,79,80], in which up to 72 N-glycans were identified by LC-MS and MALDI-TOF-MS. In this work, the many intermediate steps necessary to separately analyze free-, N- and O-glycans are most likely the reason why less N-glycans were detected compared to the number obtained by Cho et al. [59]. 

CSF free-glycans derived from glycoproteins consisted of complete, truncated N- or O-glycans as well as free monosaccharides. Interestingly, free N-glycans comprised solely high-mannoses bearing one or two GlcNAc at the reducing end. Free-glycans have been reported to be present in various body fluids, including urine [65,66,67], serum [63,64] and seminal plasma [68]. Over 130 free-glycans were reported in the urine of patients with cancer and controls: they were proposed by Hanzawa and coworkers as cancer biomarkers [65]. They consisted of free intact and truncated N-glycans, mucin-type N-glycans as well as small glycans having either lactose or N-acetyl lactosamine cores [65]. In human serum and urine, free N-glycans were comprised solely of neutral and sialylated complex-type N-glycans [63,64,65,66,67]. In rat serum, traces of high-mannose free N-glycans bearing either one or two GlcNAc at the reducing end were detected too [63]. The differences in composition between the different body fluids suggest that the glycobiological degradation processes occurring in the brain are different from the ones occurring in the rest of the body. The exact mechanisms have only been partially studied. Free-glycans are present in the cytosol in mammalian cells before they are transported to the lysosome, where they are degraded [81]. They result from the cleavage of glycoproteins in two ways. They can either be released from the dolichol-linked precursor via the action of an oligosaccharyltransferase in the endoplasmic reticulum [81,82]. Alternatively, N-Glycanase releases glycans from misfolded glycoproteins that are retrotranslocated from the endoplasmic reticulum to the cytosol [83,84]. As the free-glycan biomarkers (Hex_1_ and HexNAc_1_Hex_1_Neu5Ac_1_) that we proposed correlated with the CSF parameters, AD could be modifying the process of free-glycan formation. 

The N-glycan spectra obtained in this work for CSF are comparable with previous studies [59,77]. Similar to the brain N-glycome, they mostly consist of high-mannose, biantennary bisecting core-fucosylated N-glycans [85]. A low degree of sialylation was observed too: about 20% in CSF N-glycans and less than 10% in brain N-glycans [85]. The Alzheimer’s disease profile 1 shown by Palmigiano et al. shows the greatest similarities with our work in terms of intensities and distributions. The most abundant N-glycan in our profile at *m*/*z* 2081.1 corresponds to a core-fucosylated bisecting agalactosylated asialylated biantennary N-glycan that was previously identified in human transferrin as “brain-type transferrin” N-glycan, accounting for about 5% of transferrin isoforms [86]. Contrary to “serum-type transferrin”, the major isoform of transferrin in the CSF, “brain-type transferrin”, is synthesized within the brain and does not stem from human blood. The truncation of its galactoses and N-acetyl neuraminic acids was previously observed in rat brain as well [87]. As a result, it was postulated to result from a low expression of β 1,4 galactosyltransferase in the brain [87]. The core-fucosylated agalactosylated asialylated biantennary N-glycan at *m*/*z* 2081.1 was also found in other CSF glycoproteins such as β-site APP-cleaving enzyme-1 (BACE1) [88] and prostaglandin D synthetase [76].

The major N-glycans found in the CSF profiles in this work, namely at *m*/*z* 2018.1, 2285.2, and 2459.2, contain bisecting GlcNAc, which is a hallmark of neural glycosylation [54,59,77,89,90]. Glycoprotein bisection regulates the function of many key neural proteins via GnT-III [89]. For instance, GnT-III-deficient AD model mice showed that the lack of bisection directs BACE1 to late endosomes/lysosomes, where it is less colocalized with APP. This leads to accelerated lysosomal degradation and reduced amyloid-β (Aβ) accumulation in the brain by suppressing the function of the BACE1, a key Aβ-generating enzyme [88,89]. In parallel, a decrease in Aβ_1–40_ and Aβ_1–42_ (Aβ) was measured in vitro in GnT-III-transfected cells [53]. Cell culture experiments indicated that recombinant APP also exhibits core-fucosylated bisecting N-glycans [54]. These N-glycans were shown to have a protective function by preventing the formation of Aβ-peptides [53]. Interestingly, an overexpression of MGAT3, the gene responsible for the expression of GnT-III in the brain of AD patients, was previously reported [54]. This suggests an adaptive response to protect the brain from further β-amyloid production. N-Glycan bisection in CSF has previously been correlated with the tau protein [91]. The tau protein is originally a non-N-glycosylated cytosolic protein in healthy controls that is N-glycosylated in AD, resulting in hyperphosphorylation and aggregation [92,93]. Our data (Supplementary Material Table S10) showed a correlation between tau and bisected N-glycans, which is in line with the findings of Schedin-Weiss et al. [91].

Besides bisection, sialylation is also an important neuronal glycosylation feature. A reduced sialic acid content in free-glycan fragments and O-glycans was found in patients with AD patients compared to healthy patients. This is in line with the decrease in sialyltransferase concentrations measured in the serum of patients with AD compared to control groups as well as in post-mortem AD brains compared with controls [56,57]. BACE-1 is decisively involved in this reduced ST concentration: BACE-1 is not only an APP-processing enzyme but also cleaves β-galactoside α2,6-sialyltransferase (ST6Gal-I), thereby downregulating its transferase activity [55,58,94]. 

## 5. Conclusions

For the first time, the complete CSF glycome of patients with AD was examined and compared with controls as well as patients suffering from other types of dementia. In addition, N- and O-glycans were profiled for large cohorts and the study of free-glycans in CSF was a novel aspect of this work. We could show that two of the free-glycans had satisfactory ROC curves, demonstrating the biomarker potential for AD. In connection with this, further experiments need to be carried out to investigate the biomarker potential of free-glycans in more detail. Large-scale studies should be carried out in the form of multi-center studies. Not only should the different genders be taken into account but also other possible influencing factors, such as pre-existing or concomitant diseases, lifestyles, and genetic dispositions, should be considered.

## Figures and Tables

**Table 1:** Baseline characteristics of patients with AD, disease control patients and healthy controls. MMSE: Mini-Mental State Examination; Aβ-ratio: ratio Aβ_1–42_/Aβ_1–40_; p-tau: phosphorylated tau; t-tau: total tau protein, SD: standard deviation.

	Patients with AD	Disease Control Patients	Healthy Controls
Number of patients (m/f)	89 (43/46)	86 (55/31)	87 (48/39)
Age of the patient cohorts (years)			
Mean (m/f)	76 (75/76)	73 (72/75)	66 (67/65)
Median (m/f)	77 (77/77)	75 (72/77)	66 (66/65)
SD	6.7	8.3	8.5
Range	44–88	52–89	45–84
MMSE			
Mean (m/f)	23.5 (23.6/23.5)	23.6 (24.2/22.5)	28.7 (28.8/28.7)
Median (m/f)	24.0 (25.0/24.0)	25.0 (25.0/23.5)	29.0 (29.0/29.0)
SD	3.8	4.6	1.1
Range	12.0–30.0	3.0–30.0	26.0–30.0
Aβ ratio			
Mean (m/f)	0.051 (0.049/0.053)	0.085 (0.085/0.085)	0.088 (0.090/0.086)
Median (m/f)	0.049 (0.048/0.051)	0.095 (0.091/0.097)	0.096 (0.097/0.094)
SD	0.012	0.024	0.020
Range	0.025–0.103	0.027–0.120	0.034–0.115
p-tau (pg/mL)			
Mean (m/f)	104.0 (112.0/96.4)	53.1 (53.3/52.6)	40.9 (41.2/40.6)
Median (m/f)	98.1 (105.3/94.9)	41.9 (42.0/40.6)	37.2 (38.5/32.1)
SD	42.9	37.4	14.4
Range	34.0–279.0	24.0–253.0	17.0–91.0
t-tau (pg/mL)			
Mean (m/f)	674.6 (724.8/627.7)	385.3 (381.5/391.9)	282.8 (290.7/273.0)
Median (m/f)	615.0 (625.0/612.5)	308.0 (297.0/320.0)	241.0 (267.5/237.0)
SD	260.6	235.6	116.7
Range	231.0–1505.0	114.0–1349.0	96.0–583.0

**Table 2:** Mean relative areas and standard deviations of free-glycans isolated from the three patient cohorts as judged by MALDI-TOF-MS. AD: Alzheimer’s disease, DC: disease control, HC: healthy control. *p* values were determined using the Mann–Whitney U test. *p*-values ≤ 0.05 were considered as statistically significant and *p*-values ≤ 0.01 as highly significant.

*m*/*z*	Composition	Patients with AD [%]	DC Patients [%]	HC Patients [%]	*p* Value AD/DC	*p* Value AD/HC	*p* Value DC/HC
273.1	Hex_1_	16.65 ± 5.27	11.46 ± 5.68	10.09 ± 3.95	≤0.01	≤0.01	
314.2	HexNAc_1_	10.73 ± 2.40	11.61 ± 2.66	10.40 ± 2.68	≤0.05		≤0.01
430.2	Neu5Ac_1_	19.08 ± 3.32	18.60 ± 3.22	18.77 ± 4.24			
518.3	Hex_1_HexNAc_1_	5.62 ± 0.78	6.93 ± 1.04	6.68 ± 1.27	≤0.01	≤0.01	
559.3	HexNAc_2_	1.19 ± 0.28	1.54 ± 0.49	1.31 ± 0.37	≤0.01	≤0.05	≤0.01
675.3	Neu5Ac_1_HexNAc_1_	0.83 ± 0.14	1.12 ± 0.25	0.96 ± 0.18	≤0.01	≤0.01	≤0.01
722.4	Hex_2_HexNAc_1_	3.98 ± 0.61	4.94 ± 0.83	4.39 ± 0.96	≤0.01	≤0.01	≤0.01
733.4	Fuc_1_HexNAc_2_	1.73 ± 0.27	2.06 ± 0.49	2.07 ± 0.47	≤0.01	≤0.01	
791.4	Neu5Ac_2_	3.71 ± 2.26	3.45 ± 2.07	4.46 ± 3.02			
825.4	Fuc_2_Hex_2_	0.95 ± 0.35	1.11 ± 0.49	1.05 ± 0.34	≤0.05	≤0.05	
879.4	Neu5Ac_1_Hex_1_HexNAc_1_	6.49 ± 1.13	7.38 ± 1.28	7.69 ± 0.97	≤0.01	≤0.01	≤0.05
926.5	Hex_3_HexNAc_1_	4.34 ± 0.76	5.17 ± 0.98	5.06 ± 1.15	≤0.01	≤0.01	
967.5	Hex_2_HexNAc_2_	0.91 ± 0.15	1.04 ± 0.20	1.05 ± 0.26	≤0.01	≤0.01	
1130.6	Hex_4_HexNAc_1_	2.46 ± 0.46	2.81 ± 0.60	2.89 ± 0.74	≤0.01	≤0.01	
1171.6	Hex_3_HexNAc_2_	1.03 ± 0.20	1.08 ± 0.22	1.17 ± 0.31		≤0.01	
1212.6	Hex_2_HexNAc_3_	2.06 ± 0.53	2.24 ± 0.70	2.07 ± 0.52			
1240.6	Neu5Ac_2_Hex_1_HexNAc_1_	8.05 ± 2.16	8.17 ± 2.00	8.77 ± 1.87			
1334.7	Hex_5_HexNAc_1_	1.08 ± 0.26	1.17 ± 0.29	1.28 ± 0.39		≤0.01	
1601.8	Neu5Ac_3_Hex_1_HexNAc_1_	1.29 ± 0.52	0.95 ± 0.49	1.35 ± 0.55	≤0.01		≤0.01
Other		7.81	7.18	8.51			

**Table 3:** Statistical differences obtained between the three cohorts (AD: Alzheimer´s disease, DC: disease control, HC: healthy controls) for N-glycan relative intensities. *p*-values ≤ 0.05 were considered as statistically significant and *p*-values ≤ 0.01 as highly significant as judged from the Mann–Whitney U test.

		Male Patients			Female Patients		
*m*/*z*	Composition	AD/DC	AD/HC	DC/HC	AD/DC	AD/HC	DC/HC
1579.8	Hex_5_HexNAc_2_		≤0.05	≤0.01			
1661.8	Hex_3_HexNAc_4_				≤0.01		
1783.9	Hex_6_HexNAc_2_		≤0.05				
1835.9	Fuc_1_Hex_3_HexNAc_4_				≤0.01		≤0.05
2040.0	Fuc_1_Hex_4_HexNAc_4_				≤0.05		
2081.1	Fuc_1_Hex_3_HexNAc_5_			≤0.01			
2111.1	Hex_4_HexNAc_5_				≤0.01		≤0.05
2214.1	Fuc_2_Hex_4_HexNAc_4_	≤0.01		≤0.05			
2244.1	Fuc_1_Hex_5_HexNAc_4_			≤0.05		≤0.01	
2431.2	Neu5Ac_1_Hex_5_HexNAc_4_				≤0.05		
2459.2	Fuc_2_Hex_4_HexNAc_5_	≤0.05	≤0.01	≤0.01	≤0.05	≤0.05	≤0.01
2489.3	Fuc_1_Hex_5_HexNAc_5_				≤0.05		
2592.3	Fuc_3_Hex_5_HexNAc_4_	≤0.05					
2605.3	Neu5Ac_1_Fuc_1_Hex_5_HexNAc_4_	≤0.01	≤0.05		≤0.01	≤0.01	
2646.3	Neu5Ac_1_Fuc_1_Hex_4_HexNAc_5_	≤0.05	≤0.01		≤0.01	≤0.01	
2837.4	Fuc_3_Hex_5_HexNAc_5_				≤0.05		≤0.05
2850.4	Neu5Ac_1_Fuc_1_Hex_5_HexNAc_5_		≤0.05		≤0.05	≤0.01	
2966.5	Neu5Ac_2_Fuc_1_Hex_5_HexNAc_4_		≤0.01			≤0.01	

## Data Availability

The datasets generated for this study are available on request to the corresponding author.

## Supplementary Materials

The following supporting information can be downloaded at: https://www.mdpi.com/article/10.3390/biom14050512/s1, Figure S1: MALDI-TOF/TOF mass spectra of the [M + Na]^+^ molecular ions at *m*/*z* (a) 722.4, (b) 825.4, (c) 879.4, (d) 926.5, (e) 967.5, (f) 1130.6, (g) 1171.6, (h) 1212.6, (i) 1240.6, (j) 1334.7 derived from free glycans isolated from CSF. Registered fragments may be formed by different fragmentation pathways, only one of which is depicted in the figure. Green circle, Man; yellow circle, Gal; yellow square, GalNAc; blue square, GlcNAc; red triangle, Fuc; Neu5Ac: pink diamond. Figure S2: MALDI-TOF/TOF mass spectra of the [M + Na]^+^ molecular ions at *m*/*z*. Figure S3: Inter-day reproducibility of the analytical workflow used to prepare. Table S1: Two-way analysis of variance (ANOVA) to assess any statistical significance of gender in the dataset of freeglycans. Table S2: Two-way analysis of variance (ANOVA) to evaluate the statistical significance of gender in the dataset of Nglycans. Table S3: Two-way analysis of variance (ANOVA) to evaluate the statistical significance of gender in the dataset of Oglycans. Table S4: N-Glycans detected in this study by MALDI-TOF-MS. Table S5: ROC curves were generated for free glycans using 89 patients with AD. 86 disease control patients and 87 healthy control patients. Table S6: Correlations were calculated between the free glycans Hex1 (*m*/*z* 273.1), HexNAc1Hex1Neu5Ac1 (*m*/*z* 879.4) and CSF parameters using the using the Spearman’s rank correlation coefficient. Table S7: Mean relative areas and standard deviations of N-glycans isolated from the three patient cohorts as judged by MALDI-TOF-MS. Table S8: Mean relative areas and standard deviations of O-glycans isolated from the three patient cohorts as judged by MALDI-TOF-MS. Table S9: Glycan types and Glycosylation features used to generate the mean relative intensities presented in Figure 3. Table S10: Correlations were calculated between N-glycans groups by features and t-Tau using the Spearman´s rank.
