# Supplementary material for: The Cerebrospinal Fluid Free-Glycans Hex1 and HexNAc1Hex1Neu5Ac1 as Potential Biomarkers of Alzheimer’s Disease
Source: Biomolecules. 2024 Apr 24;14(5):512. doi: 10.3390/biom14050512 (PMC11117705; doi:10.3390/biom14050512)
Supplement: Supplementary file 1 [file biomolecules-14-00512-s001.zip › biomolecules-2739048-supplementary.pdf]

(a)

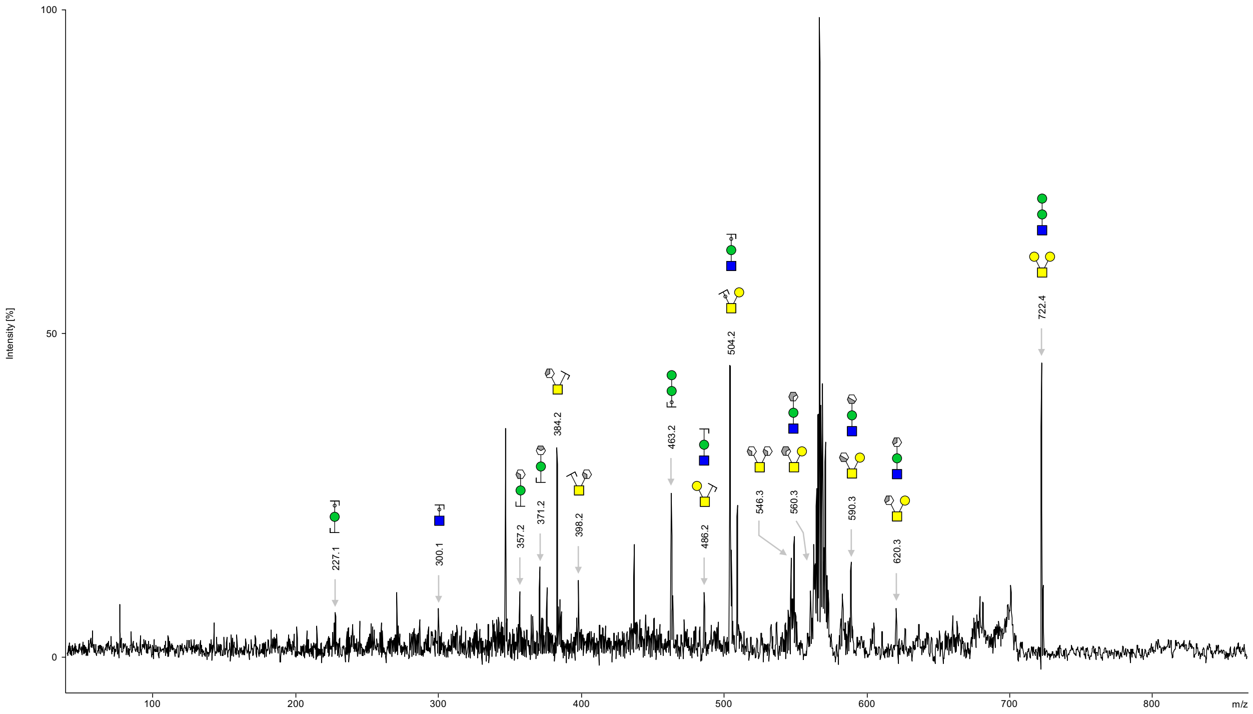

Intensity [%]
100
50
0
227.1
300.1
357.2
371.2
384.2
398.2
463.2
486.2
504.2
546.3
560.3
590.3
620.3
722.4
100
200
300
400
500
600
700
800
m/z

(b)

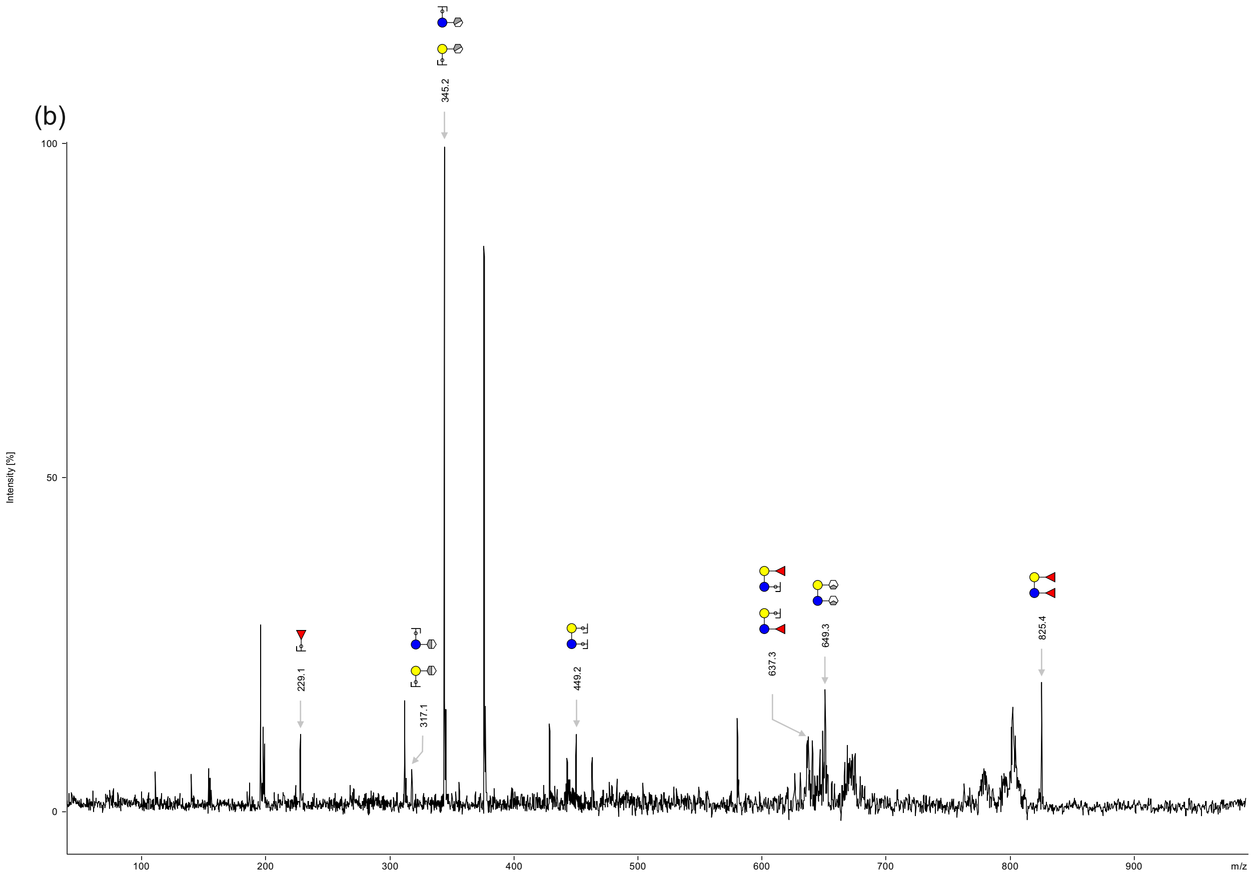

Intensity [%]
100
50
0
229.1
317.1
345.2
449.2
637.3
649.3
825.4
100
200
300
400
500
600
700
800
900
m/z

(c)

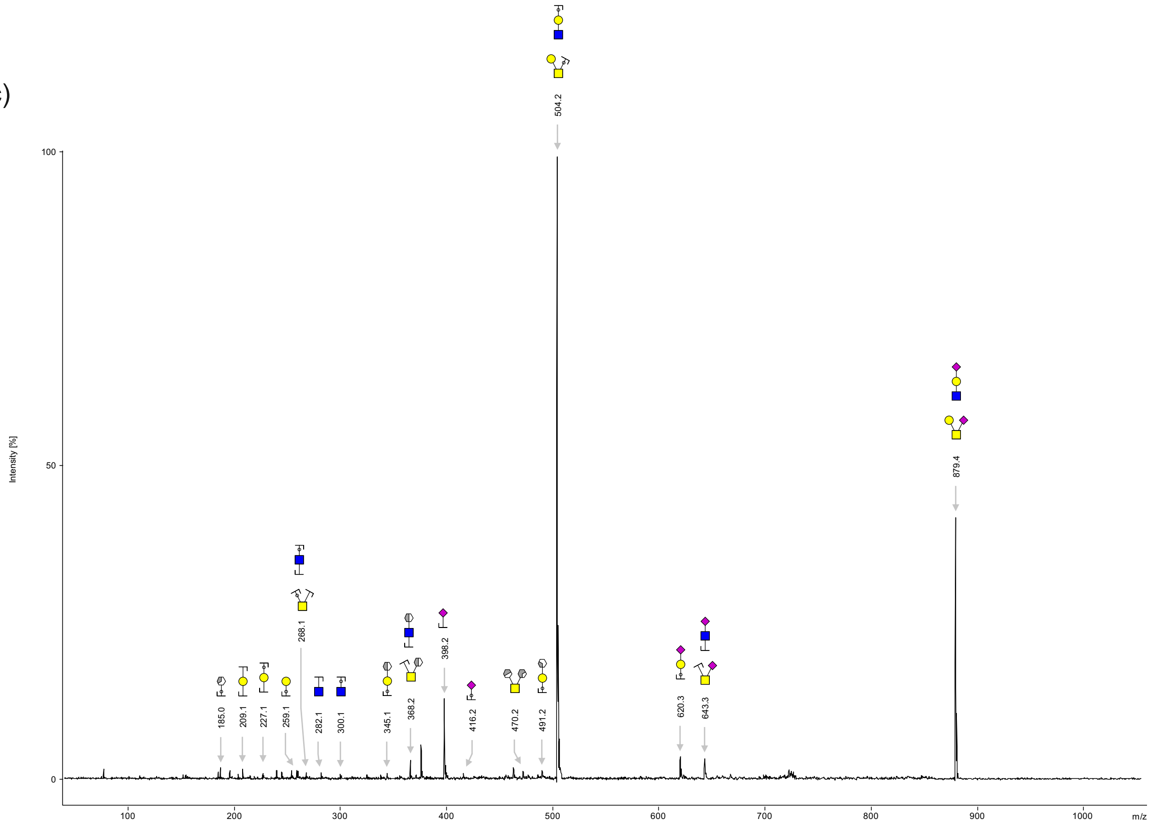
Intensity [%]
100
50
0
504.2
879.4
185.0
209.1
227.1
259.1
268.1
282.1
300.1
345.1
368.2
398.2
416.2
470.2
491.2
620.3
643.3
100
200
300
400
500
600
700
800
900
1000
m/z

(d)

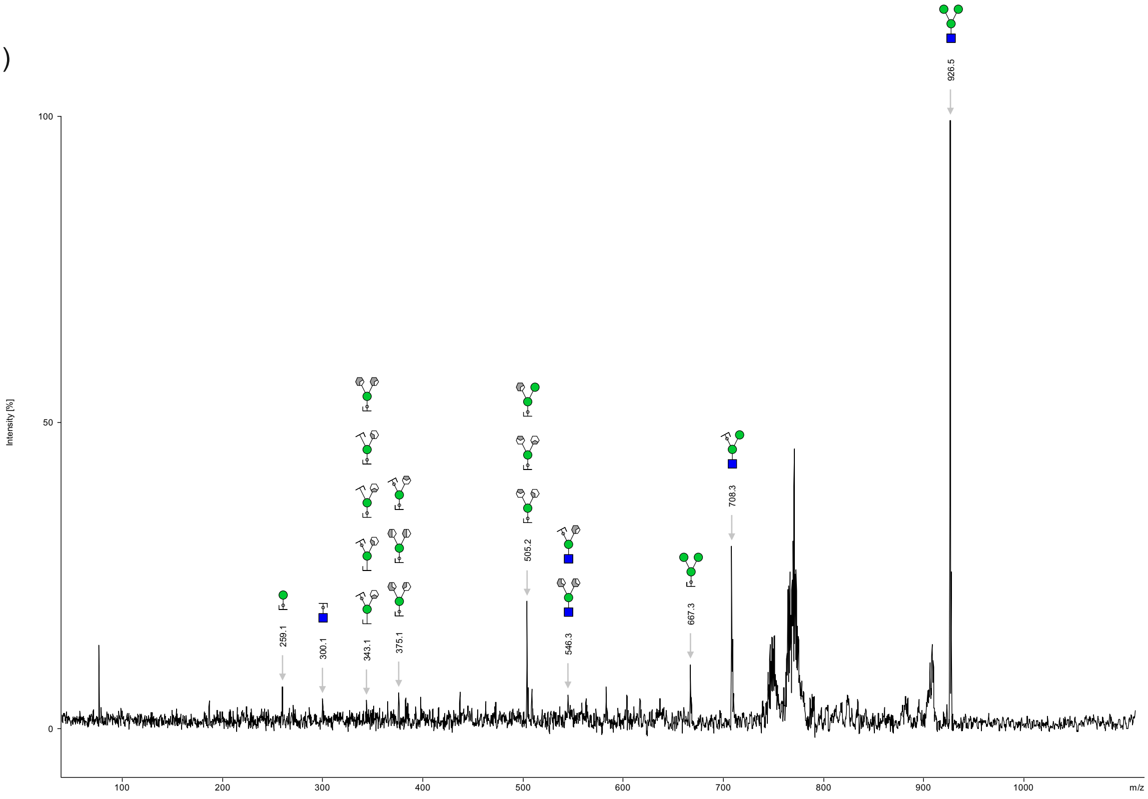
Intensity [%]
100
50
0
926.5
259.1
300.1
343.1
375.1
505.2
546.3
667.3
708.3
100
200
300
400
500
600
700
800
900
1000
m/z

(e)

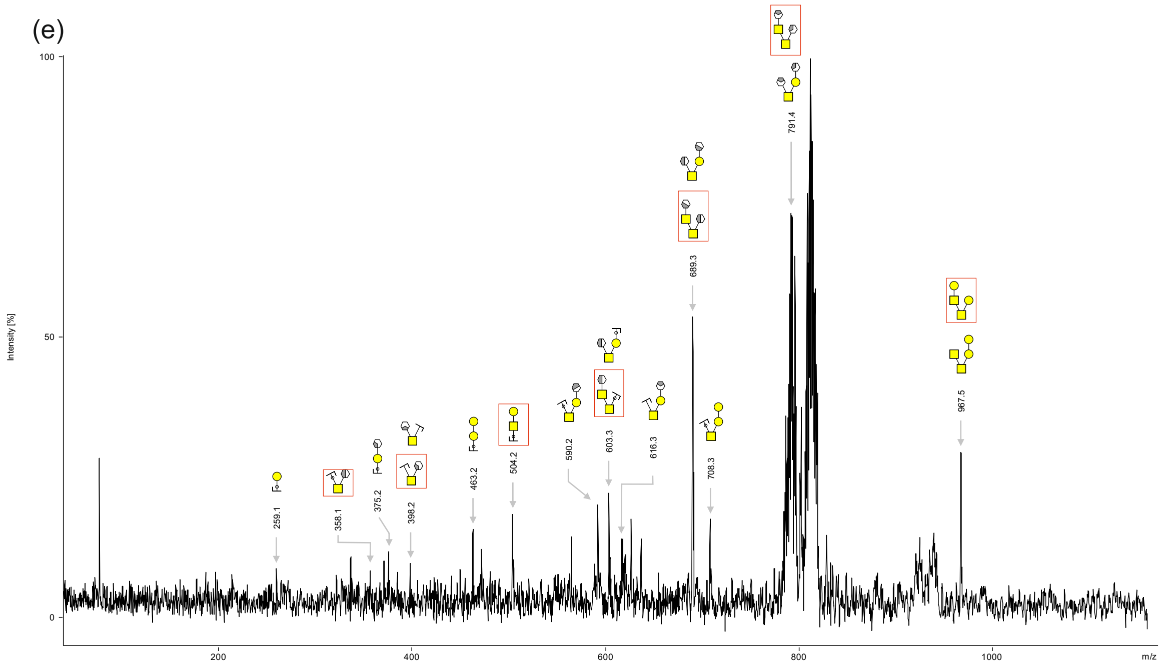

Intensity [%]
100
50
0
200
400
600
800
1000
m/z
259.1
358.1
375.2
398.2
463.2
504.2
590.2
603.3
616.3
689.3
708.3
791.4
967.5

(f)

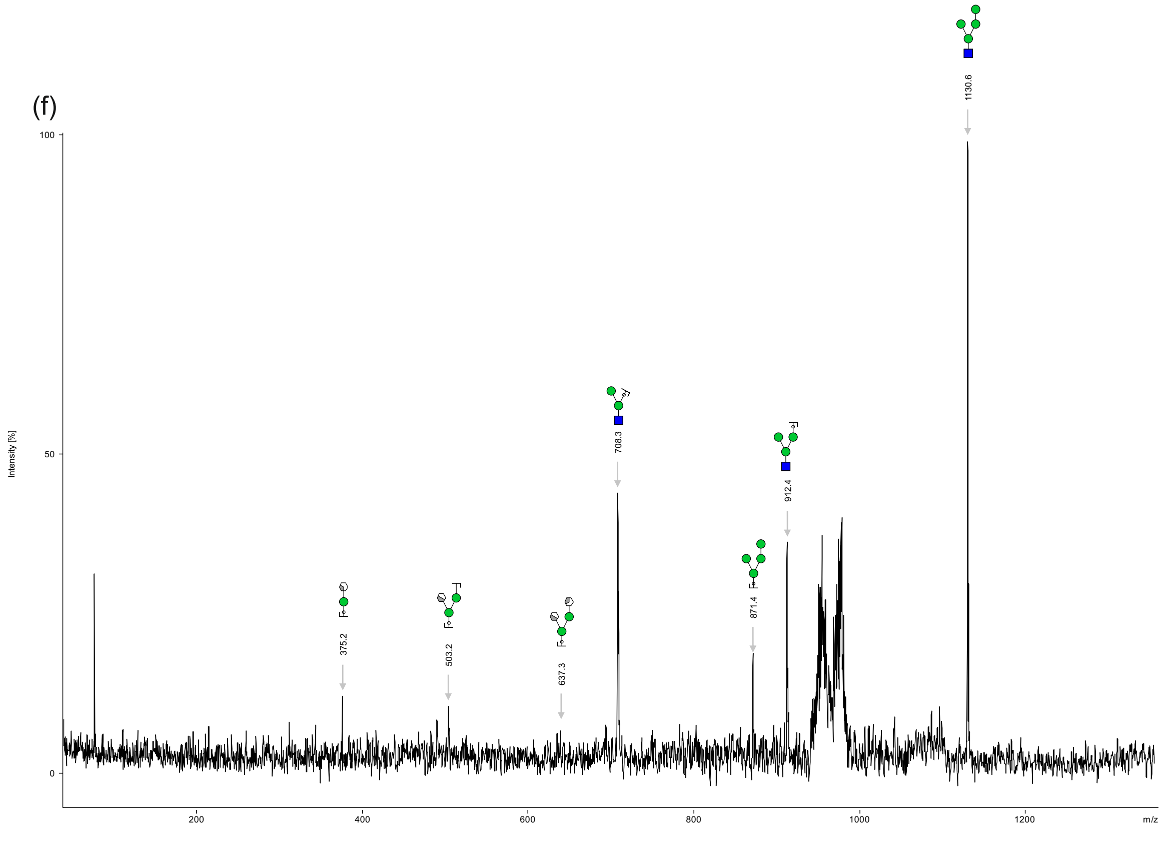

Intensity [%]
100
50
0
200
400
600
800
1000
1200
m/z
375.2
503.2
637.3
708.3
871.4
912.4
1130.6

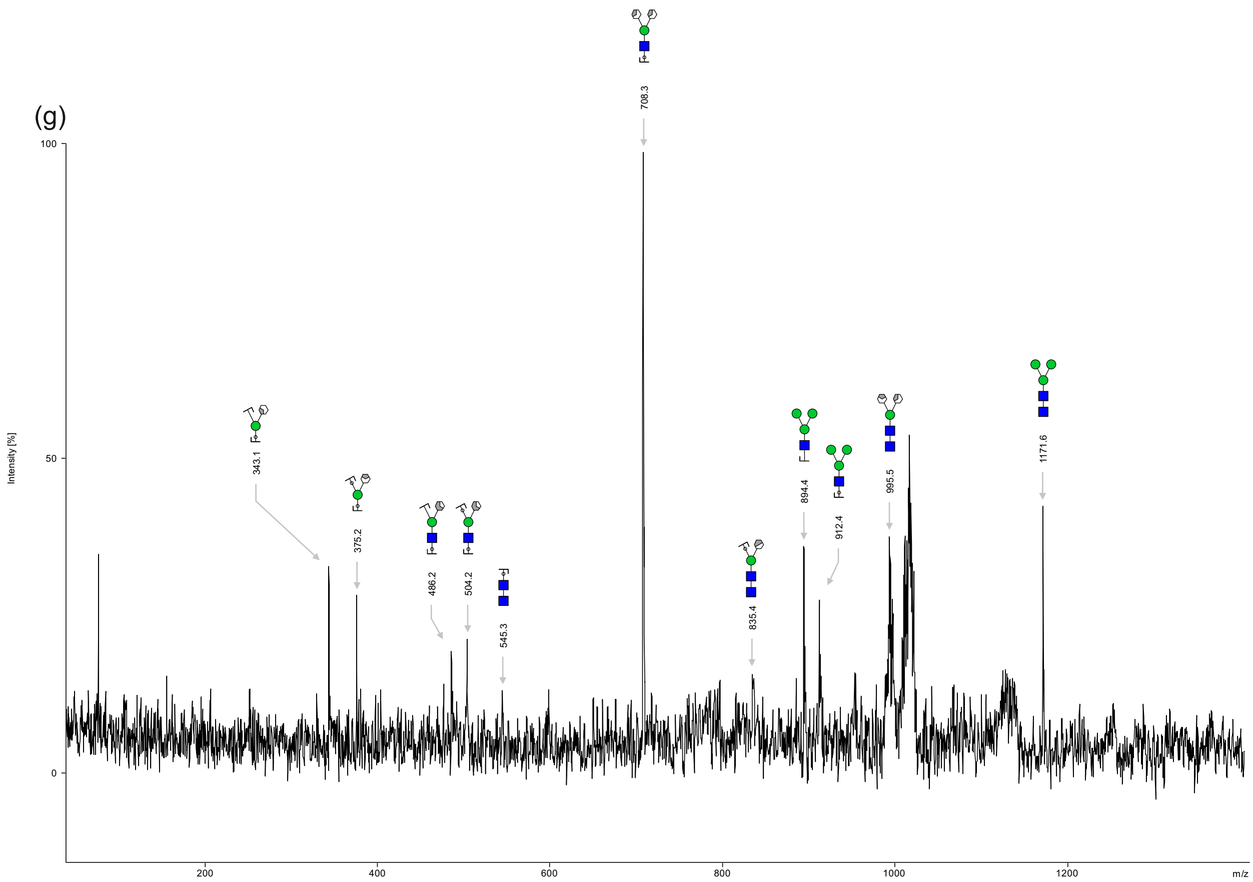
(g)
100
50
0
Intensity [%]
343.1
375.2
486.2
504.2
545.3
708.3
835.4
894.4
912.4
995.5
1171.6
200
400
600
800
1000
1200
m/z

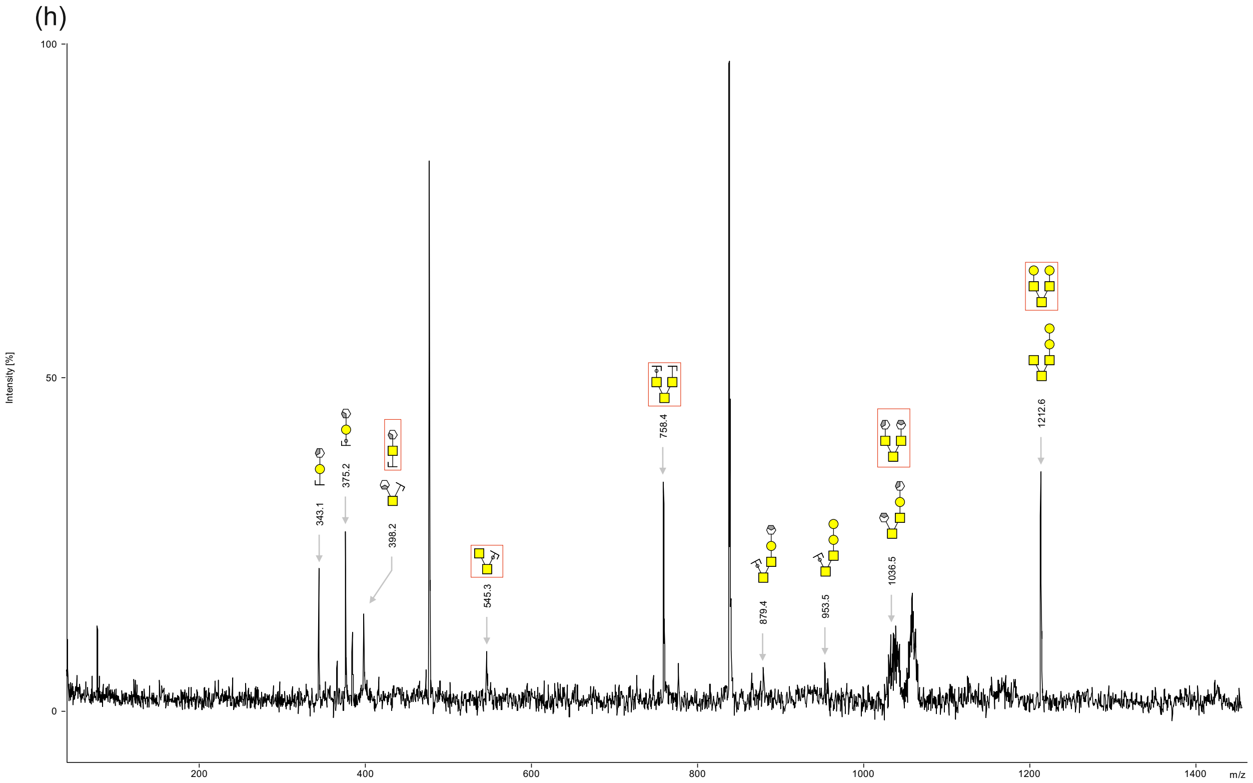
(h)
100
50
0
Intensity [%]
343.1
375.2
398.2
545.3
758.4
879.4
953.5
1036.5
1212.6
200
400
600
800
1000
1200
1400
m/z

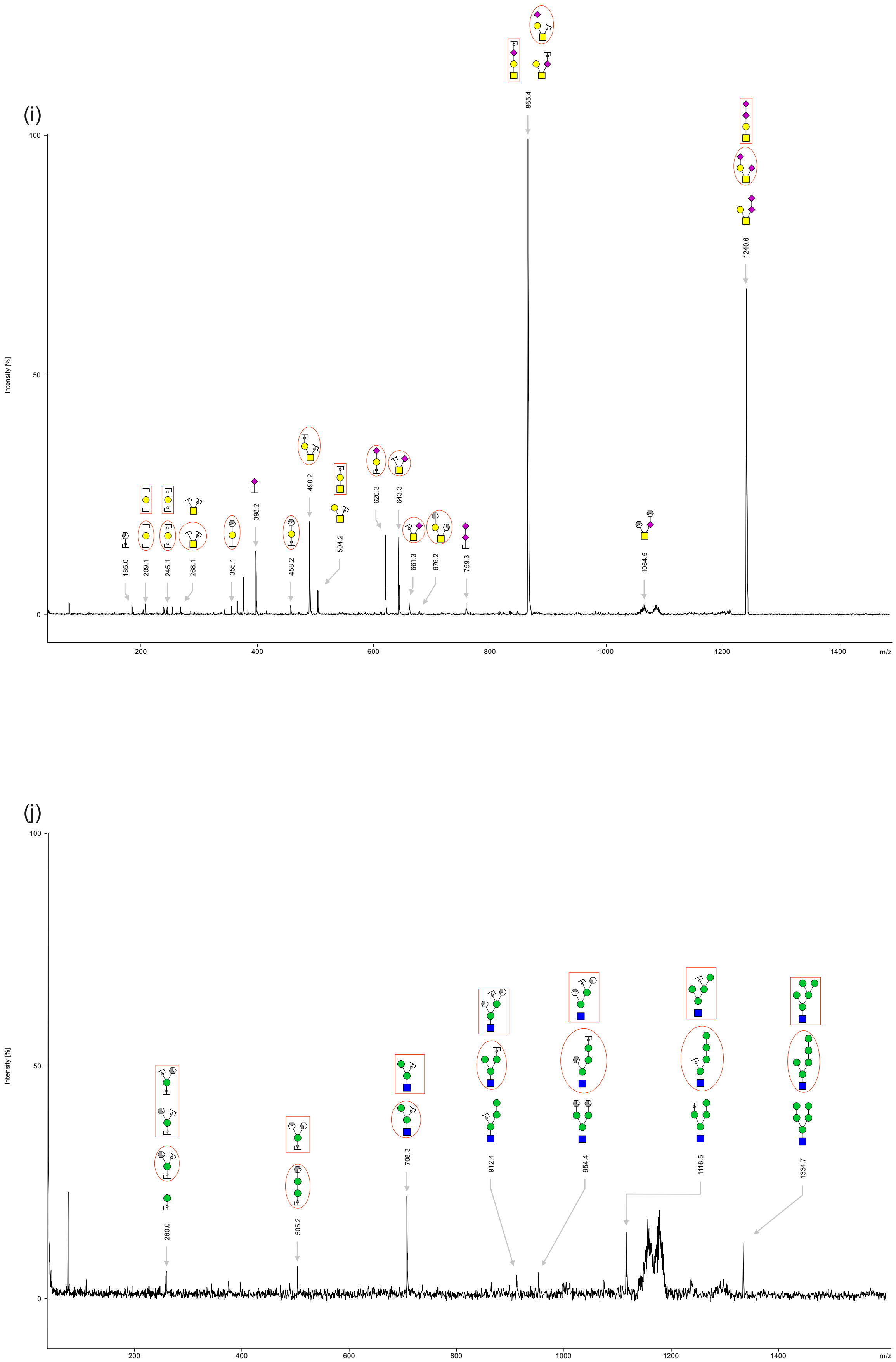

Figure S1. MALDI-TOF/TOF mass spectra of the $[M + Na]^+$ molecular ions at *m/z* (a) 722.4, (b) 825.4, (c) 879.4, (d) 926.5, (e) 967.5, (f) 1130.6, (g) 1171.6, (h) 1212.6, (i) 1240.6, (j) 1334.7 derived from free glycans isolated from CSF. Registered fragments may be formed by different fragmentation pathways, only one of which is depicted in the figure. Green circle, Man; yellow circle, Gal; yellow square, GalNAc; blue square, GlcNAc; red triangle, Fuc; Neu5Ac: pink diamond.

(a)

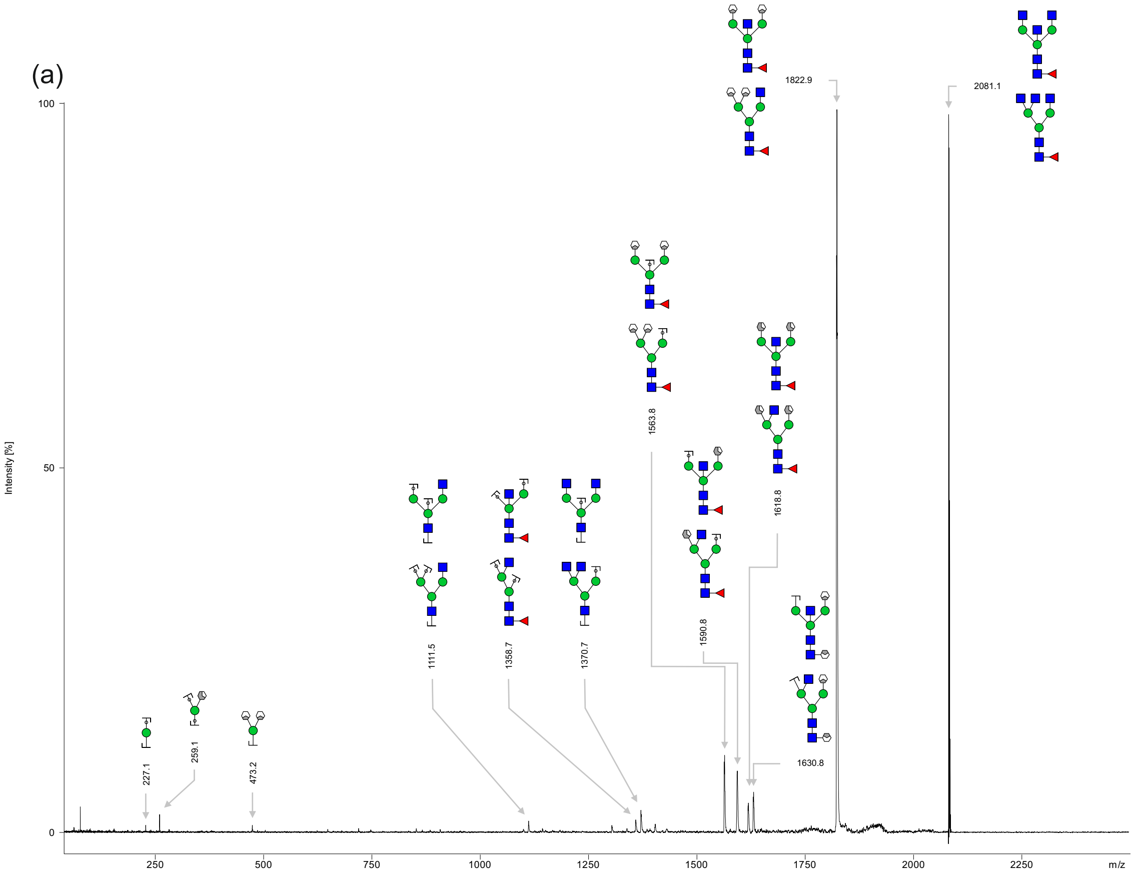

Intensity [%]
100
50
0
227.1
259.1
473.2
1111.5
1358.7
1370.7
1563.8
1590.8
1618.8
1630.8
1822.9
2081.1
250
500
750
1000
1250
1500
1750
2000
2250
m/z

(b)

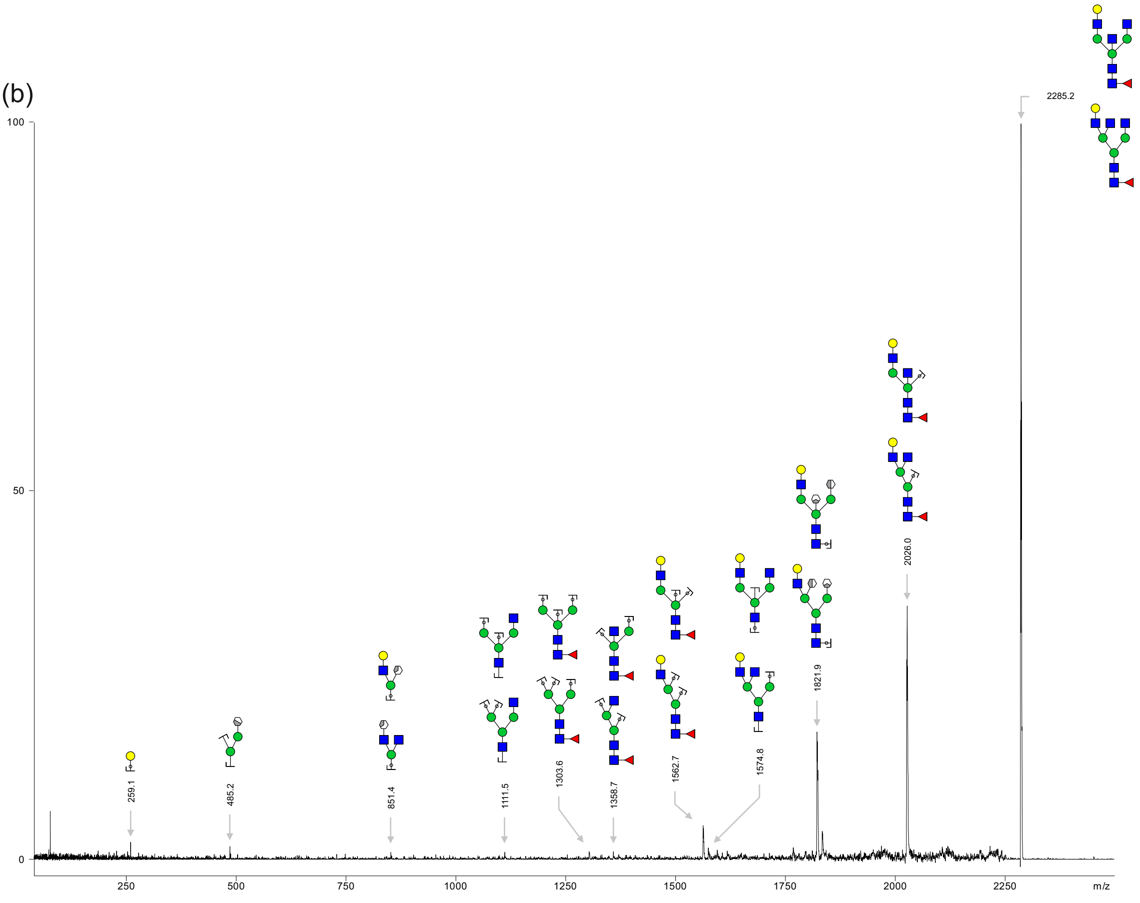

100
50
0
259.1
485.2
851.4
1111.5
1303.6
1358.7
1562.7
1574.8
1821.9
2026.0
2285.2
250
500
750
1000
1250
1500
1750
2000
2250
m/z

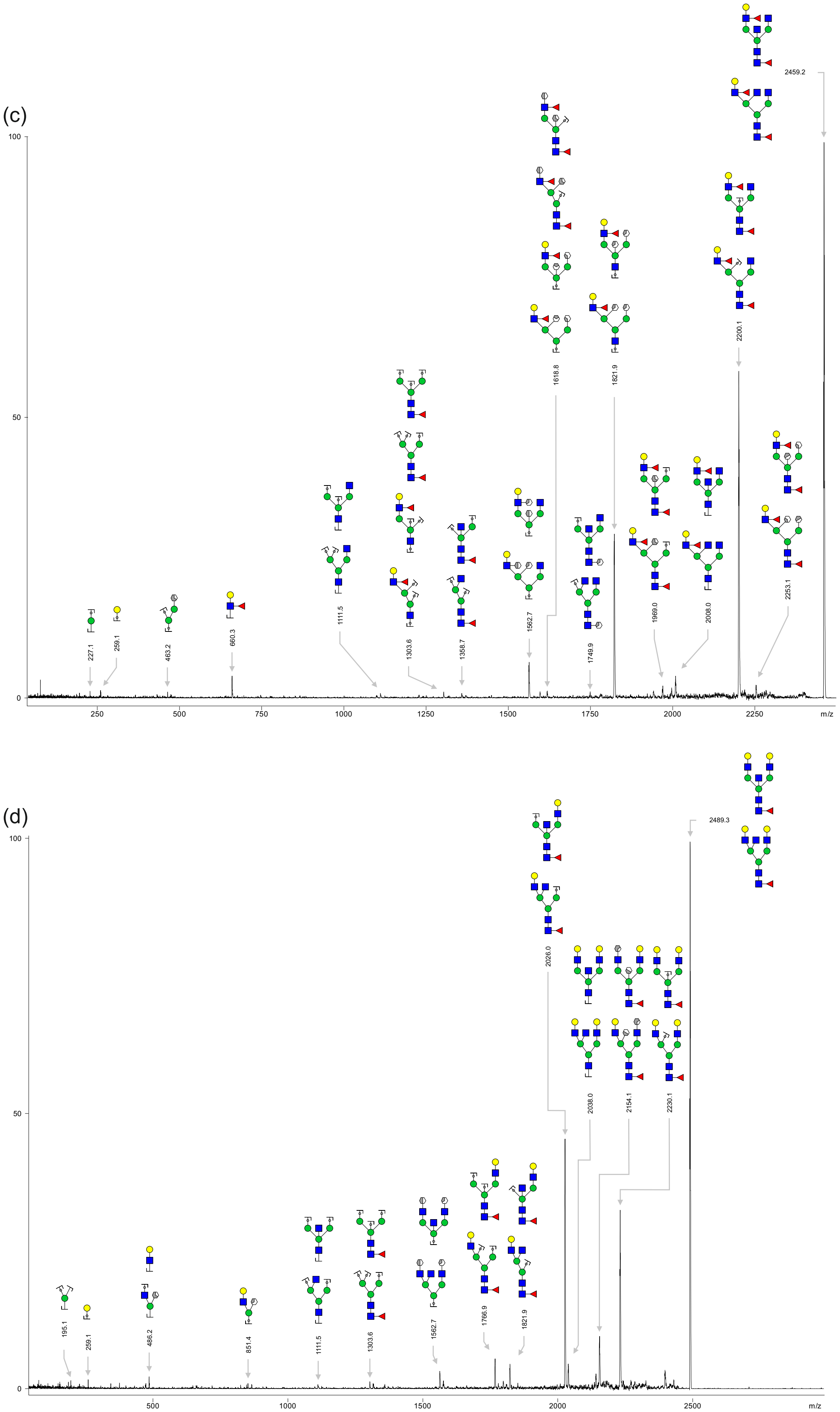
(c)
100
50
0
227.1
259.1
463.2
660.3
1111.5
1303.6
1358.7
1562.7
1618.8
1749.9
1821.9
1969.0
2008.0
2200.1
2253.1
2459.2
250
500
750
1000
1250
1500
1750
2000
2250
m/z
(d)
100
50
0
195.1
259.1
486.2
851.4
1111.5
1303.6
1562.7
1766.9
1821.9
2026.0
2038.0
2154.1
2230.1
2489.3
500
1000
1500
2000
2500
m/z

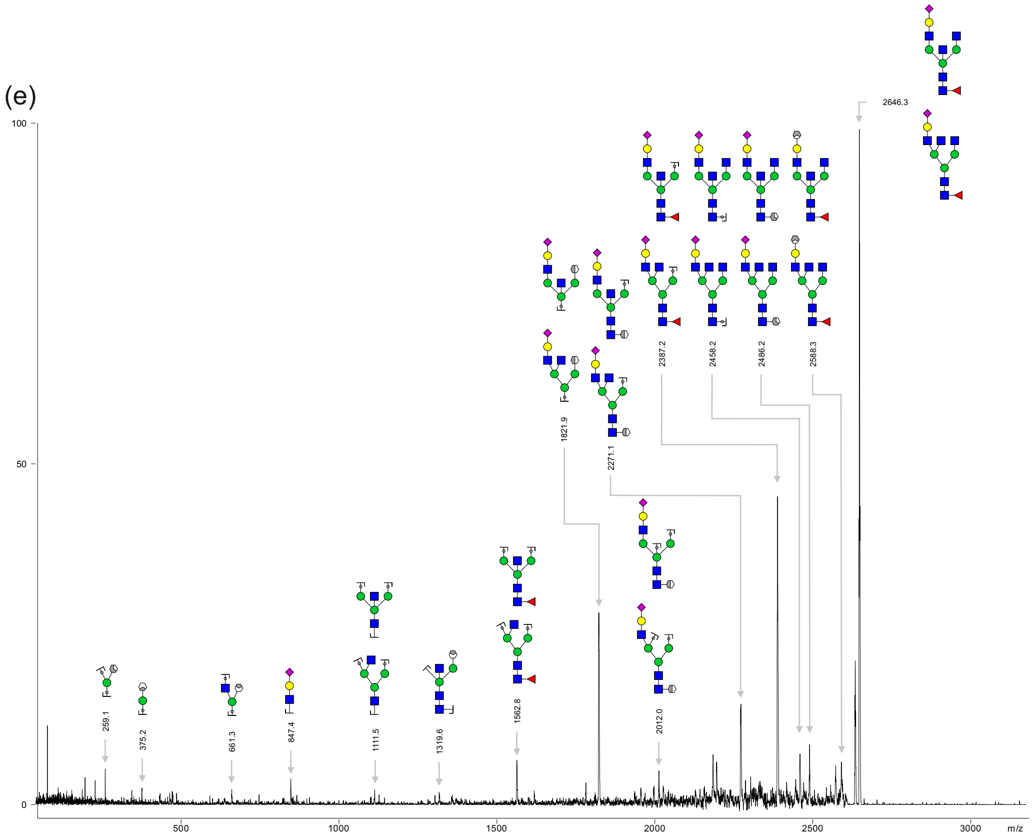
(e)
100
50
0
259.1
375.2
661.3
847.4
1111.5
1319.6
1562.8
1821.9
2012.0
2271.1
2387.2
2458.2
2486.2
2588.3
2646.3
500
1000
1500
2000
2500
3000
m/z

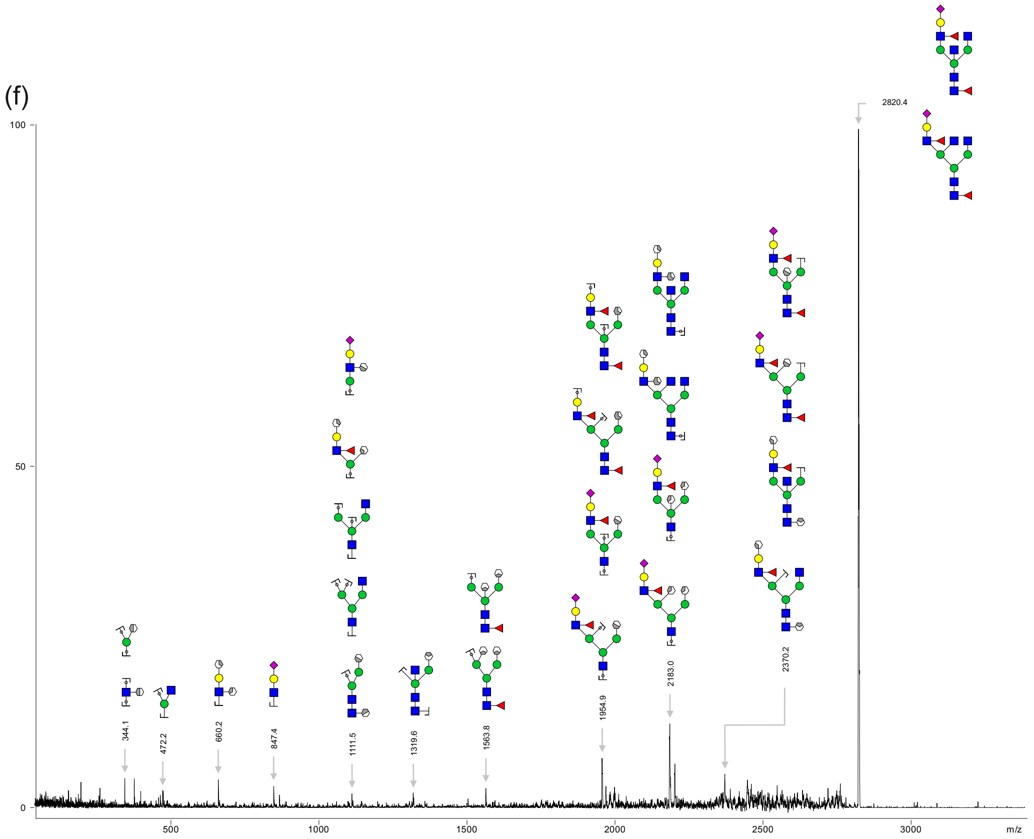
(f)
100
50
0
344.1
472.2
660.2
847.4
1111.5
1319.6
1563.8
1954.9
2183.0
2370.2
2820.4
500
1000
1500
2000
2500
3000
m/z

(g)

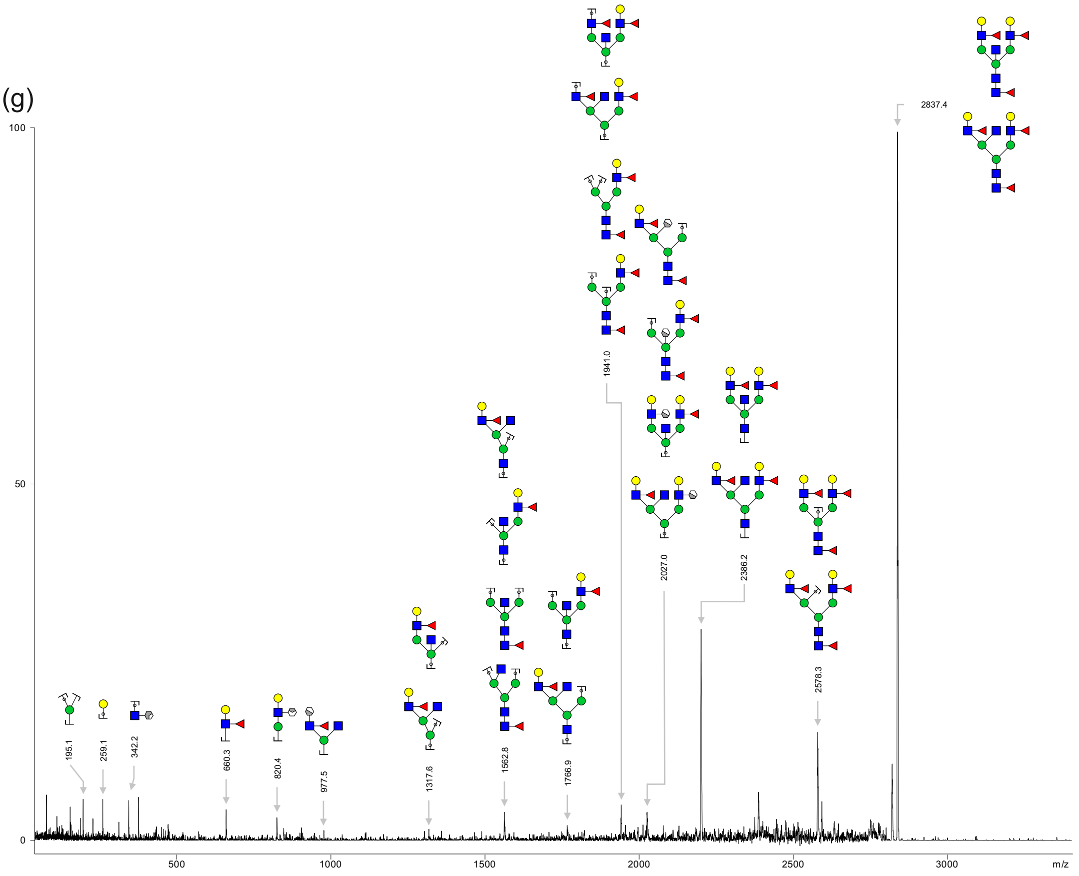

(h)

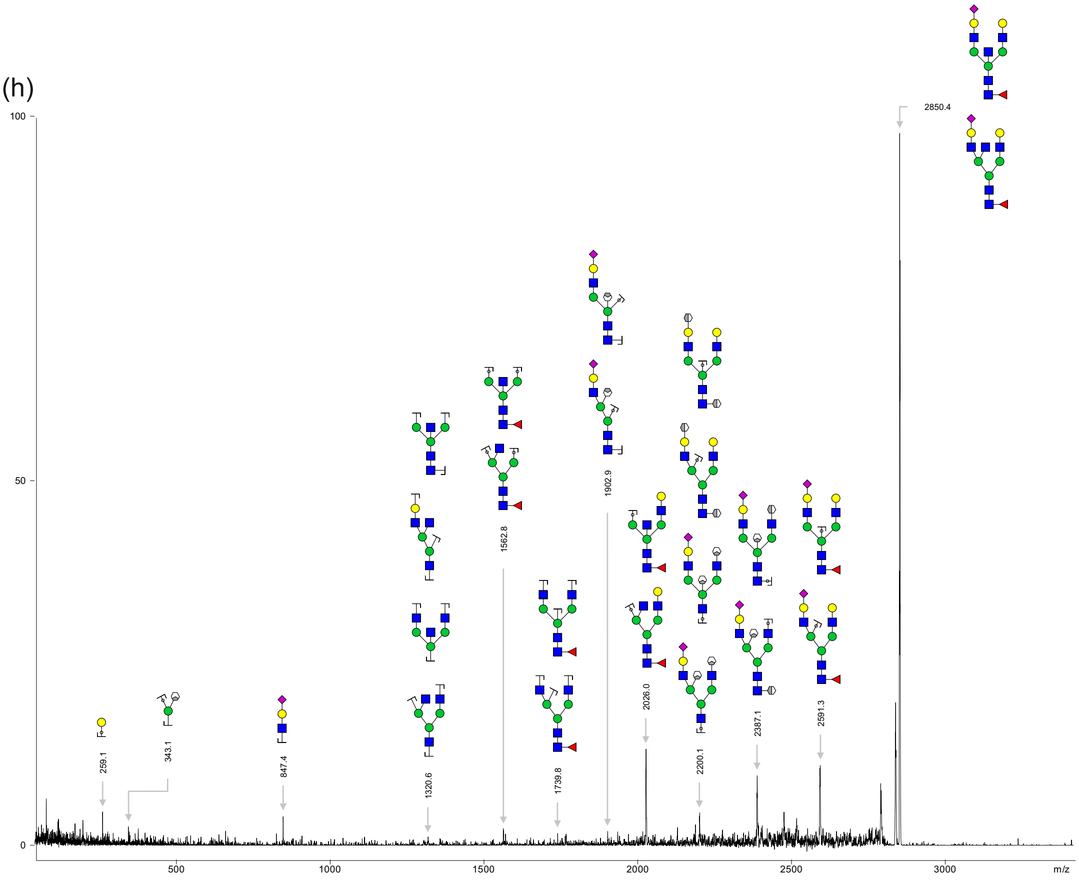

Figure S2. MALDI-TOF/TOF mass spectra of the [M + Na]+ molecular ions at m/z (a) 2081.1, (b) 2285.2 (c) 2459.2 (d) 2489.3 (e) 2646.3, (f) 2820.4 (g) 2837.4, (h) 2850.4, derived from N-glycans isolated from CSF. Registered fragments may be formed by different fragmentation pathway which is depicted in the figure. Green circle, Man; yellow circle, Gal; blue square, GlcNAc; red triangle, Fuc; Neu5Ac: pink diamond.

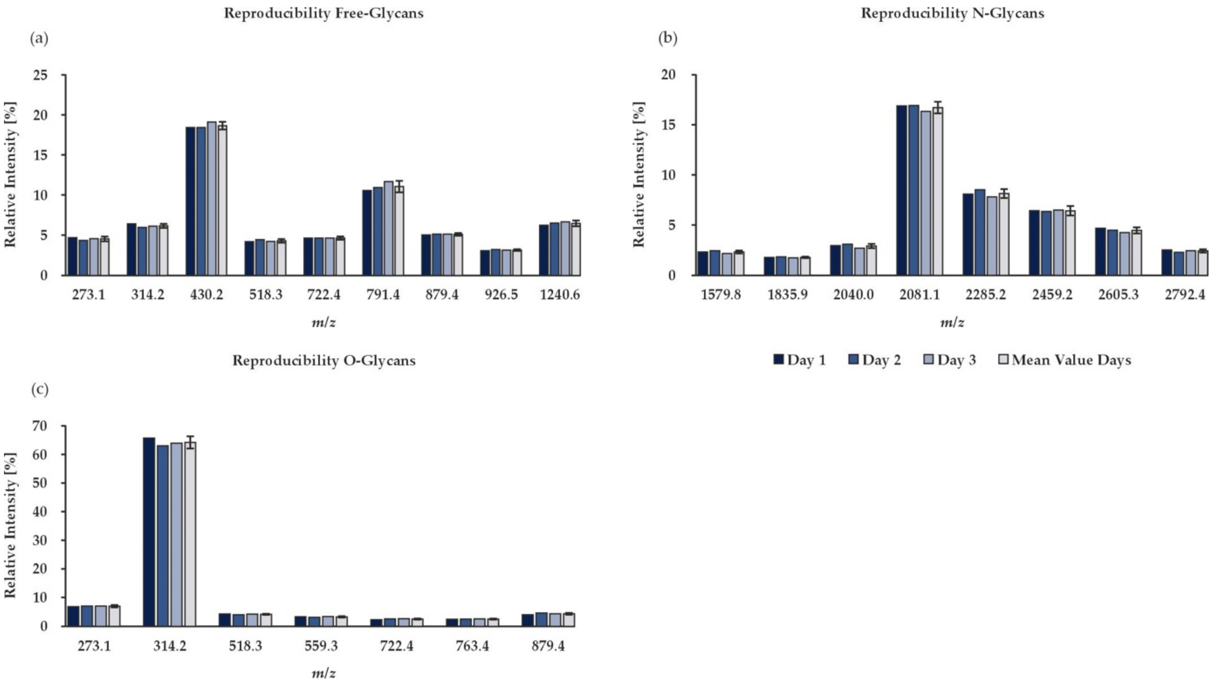

Figure S3: Inter-day reproducibility of the analytical workflow used to prepare (a) free glycans, (b) N-glycans and (c) O-glycans from CSF. Average is shown as mean ± SD.

Supplementary Material: Tables

Table S1: Two-way analysis of variance (ANOVA) to assess any statistical significance of gender in the dataset of free-glycans. P-values ≤ 0.05 were considered as statistically significant and p-values ≤ 0.01 as highly significant.

m/z	**Composition**	**Significance Levene-Test**	**Significance - Group**	**Significance – Sex**	**Significance – Group * Sex**
**273.1**	Hex_1	0.002	0.000	0.799	0.658
**314.2**	$HexNAc_1$	0.098	0.003	0.217	0.457
**430.2**	$Neu5Ac_1$	0.034	0.682	0.462	0.708
**518.3**	$Hex_1HexNAc_1$	0.000	0.000	0.546	0.262
**559.3**	$HexNAc_2$	0.001	0.000	0.165	0.276
**675.3**	$Neu5Ac_1HexNAc_1$	0.003	0.000	0.083	0.214
**722.4**	$Hex_2HexNAc_1$	0.004	0.000	0.500	0.770
**733.4**	$Fuc_1HexNAc_2$	0.009	0.000	0.482	0.289
**791.4**	$Neu5Ac_2$	0.001	0.025	0.584	0.584
**825.4**	Fuc_2Hex_2	0.031	0.033	0.901	0.974
**879.4**	$NeuAc_1Hex_1HexNAc_1$	0.209	0.000	0.733	0.364
**926.5**	$Hex_3HexNAc_1$	0.003	0.000	0.592	0.802
**967.5**	$Hex_2HexNAc_2$	0.000	0.000	0.215	0.675
**1130.6**	$Hex_4HexNAc_1$	0.000	0.000	0.919	0.954
**1171.6**	$Hex_3HexNAc_2$	0.000	0.002	0.200	0.818
**1212.6**	$Hex_2HexNAc_3$	0.006	0.143	0.238	0.857
**1240.6**	$Neu5Ac_2Hex_1HexNAc_1$	0.927	0.032	0.789	0.494
**1334.7**	$Hex_5HexNAc_1$	0.002	0.000	0.974	0.971
**1601.8**	$Neu5Ac_3Hex_1HexNAc_1$	0.930	0.000	0.451	0.516

Table S2: Two-way analysis of variance (ANOVA) to evaluate the statistical significance of gender in the dataset of N-glycans. P-values ≤ 0.05 were considered as statistically significant and p-values ≤ 0.01 as highly significant.

m/z	Composition	Significance Levene-Test	Significance Patient Groups	Significance – Sex	Significance – Group * Sex
**1579.8**	$Hex_5HexNAc_2$	0.001	0.002	0.001	0.282
**1661.8**	$Hex_3HexNAc_4$	0.007	0.000	0.357	0.030
**1783.9**	$Hex_6HexNAc_2$	0.001	0.025	0.438	0.566
**1835.9**	$Fuc_1Hex_3HexNAc_4$	0.000	0.012	0.422	0.013
**2040.0**	$Fuc_1Hex_4HexNAc_4$	0.005	0.188	0.041	0.129
**2070.0**	$Hex_5HexNAc_4$	0.002	0.506	0.780	0.937
**2081.1**	$Fuc_1Hex_3HexNAc_5$	0.000	0.006	0.023	0.109
**2111.1**	$Hex_4HexNAc_5$	0.118	0.004	0.770	0.540
**2214.1**	$Fuc_2Hex_4HexNAc_4$	0.354	0.006	0.214	0.010
**2244.1**	$Fuc_1Hex_5HexNAc_4$	0.008	0.003	0.006	0.736
**2285.2**	$Fuc_1Hex_4HexNAc_5$	0.000	0.137	0.574	0.359
**2431.2**	$Neu5Ac_1Hex_5HexNAc_4$	0.025	0.254	0.723	0.312
**2459.2**	$Fuc_2Hex_4HexNAc_5$	0.016	0.000	0.000	0.972
**2489.3**	$Fuc_1Hex_5HexNAc_5$	0.009	0.009	0.045	0.766
**2592.3**	$Fuc_3Hex_5HexNAc_4$	0.051	0.023	0.939	0.731
**2605.3**	$Neu5Ac_1Fuc_1Hex_5HexNAc_4$	0.002	0.000	0.014	0.971
**2646.3**	$Neu5Ac_1Fuc_1Hex_4HexNAc_5$	0.008	0.000	0.189	0.966
**2792.4**	$Neu5Ac_2Hex_5HexNAc_4$	0.105	0.076	0.576	0.724
**2820.4**	$Neu5Ac_1Fuc_2Hex_4HexNAc_5$	0.273	0.048	0.835	0.950
**2837.4**	$Fuc_3Hex_5HexNAc_5$	0.026	0.001	0.188	0.448
**2850.4**	$Neu5Ac_1Fuc_1Hex_5HexNAc_5$	0.003	0.000	0.977	0.657
**2966.5**	$Neu5Ac_2Fuc_1Hex_5HexNAc_4$	0.000	0.000	0.060	0.866

Table S3: Two-way analysis of variance (ANOVA) to evaluate the statistical significance of gender in the dataset of O-glycans. P-values ≤ 0.05 were considered as statistically significant and p-values ≤ 0.01 as highly significant.

m/z	Composition	Significance Levene-Test	Significance – Patient Groups	Significance – Sex	Significance – Group * Sex
**273.1**	Hex_1	0.000	0.000	0.036	0.006
**314.2**	$HexNAc_1$	0.145	0.002	0.104	0.197
**518.3**	$Hex_1HexNAc_1$	0.002	0.000	0.416	0.633
**559.3**	$HexNAc_2$	0.309	0.000	0.856	0.823
**675.3**	$NeuAc_1HexNAc_1$	0.871	0.001	0.941	0.563
**722.4**	$Hex_2HexNAc_1$	0.962	0.000	0.747	0.720
**763.4**	$Hex_1HexNAc_2$	0.383	0.000	0.987	0.777
**804.4**	$HexNAc_3$	0.132	0.000	0.668	0.436
**825.4**	Fuc_2Hex_2	0.102	0.000	0.923	0.270
**879.4**	$NeuAc_1Hex_1HexNAc_1$	0.000	0.000	0.551	0.423
**967.5**	$Hex_2HexNAc_2$	0.057	0.001	0.907	0.540
**1124.6**	$Neu5Ac_1Hex_1HexNAc_2$	0.066	0.003	0.029	0.918
**1240.6**	$NeuAc_2Hex_1HexNAc_1$	0.000	0.000	0.091	0.575

Table S4: N-Glycans detected in this study by MALDI-TOF-MS.

m/z	Composition	
1171.6	$Hex_3HexNAc_2$	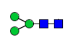
1345.7	$Fuc_1Hex_3HexNAc_2$	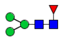
1375.7	$Hex_4HexNAc_2$	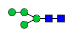
1579.8	$Hex_5HexNAc_2$	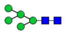
1590.8	$Fuc_1Hex_3HexNAc_3$	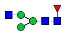
1620.8	$Hex_4HexNAc_3$	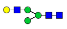
1661.8	$Hex_3HexNAc_4$	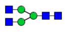
1753.9	$Fuc_1Hex_5HexNAc_2$	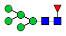
1783.9	$Hex_6HexNAc_2$	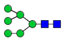
1794.9	$Fuc_1Hex_4HexNAc_3$	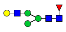
1835.9	$Fuc_1Hex_3HexNAc_4$	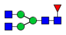
1865.9	$Hex_4HexNAc_4$	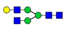
1907.0	$Hex_3HexNAc_5$	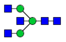
1982.0	$Neu5Ac_1Hex_4HexNAc_3$	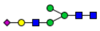
1988.0	$Hex_7HexNAc_2$	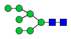
1999.0	$Fuc_1Hex_5HexNAc_3$	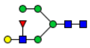
2010.0	$Fuc_2Hex_3HexNAc_5$	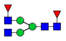
2029.0	$Hex_6HexNAc_3$	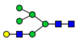
2040.0	$Fuc_1Hex_4HexNAc_4$	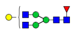
2070.0	$Hex_5HexNAc_4$	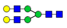
2081.1	$Fuc_1Hex_3HexNAc_5$	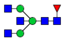
2111.1	$Hex_4HexNAc_5$	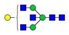
2152.1	$Hex_3HexNAc_6$	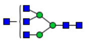
2156.1	$Neu5Ac_1Fuc_1Hex_4HexNAc_3$	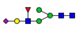
2192.1	$Hex_8HexNAc_2$	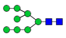
2214.1	$Fuc_2Hex_4HexNAc_4$	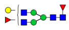
2227.1	$Neu5Ac_1Hex_4HexNAc_4$	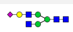
2244.1	$Fuc_1Hex_5HexNAc_4$	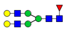
2285.2	$Fuc_1Hex_4HexNAc_5$	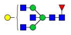
2315.2	$Hex_5HexNAc_5$	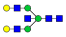
2326.2	$Fuc_1Hex_3HexNAc_6$	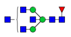
2360.2	$Neu5Ac_1Fuc_1Hex_5HexNAc_3$	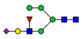
2390.2	$Neu5Ac_1Hex_6HexNAc_3$	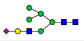
2396.2	$Hex_9HexNAc_2$	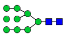
2401.2	$Neu5Ac_1Fuc_1Hex_4HexNAc_4$	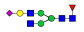
2418.2	$Fuc_2Hex_5HexNAc_4$	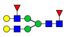
2431.2	$Neu5Ac_1Hex_5HexNAc_4$	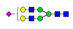
2459.2	$Fuc_2Hex_4HexNAc_5$	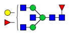

2472.2	Neu5Ac$_1$Hex$_4$HexNAc$_5$	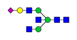
2489.3	Fuc$_1$Hex$_5$HexNAc$_5$	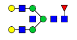
2519.3	Hex$_6$HexNAc$_5$	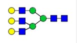
2530.3	Fuc$_1$Hex$_4$HexNAc$_6$	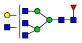
2560.3	Hex$_5$HexNAc$_6$	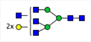
2564.3	Neu5Ac$_1$Fuc$_1$Hex$_6$HexNAc$_3$	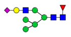
2592.3	Fuc$_3$Hex$_5$HexNAc$_4$	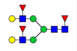
2605.3	Neu5Ac$_1$Fuc$_1$Hex$_5$HexNAc$_4$	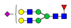
2646.3	Neu5Ac$_1$Fuc$_1$Hex$_4$HexNAc$_5$	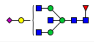
2663.3	Fuc$_2$Hex$_5$HexNAc$_5$	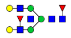
2676.3	Neu5Ac$_1$Hex$_5$HexNAc$_5$	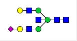
2693.4	Fuc$_1$Hex$_6$HexNAc$_5$	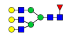
2704.4	Fuc$_2$Hex$_4$HexNAc$_6$	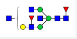
2717.4	Neu5Ac$_1$Hex$_4$HexNAc$_6$	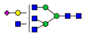
2734.4	Fuc$_1$Hex$_5$HexNAc$_6$	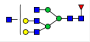
2749.5	Neu5Ac$_1$Fuc$_3$Hex$_4$HexNAc$_4$	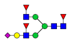
2764.4	Hex$_6$HexNAc$_6$	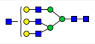
2792.4	Neu5Ac$_2$Hex$_5$HexNAc$_4$	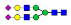
2820.4	Neu5Ac$_1$Fuc$_2$Hex$_4$HexNAc$_5$	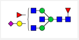
2837.4	Fuc$_3$Hex$_5$HexNAc$_5$	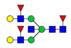
2850.4	Neu5Ac$_1$Fuc$_1$Hex$_5$HexNAc$_5$	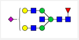
2880.4	Neu5Ac$_1$Hex$_6$HexNAc$_5$	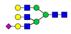
2891.5	Neu5Ac$_1$Fuc$_1$Hex$_4$HexNAc$_6$	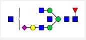
2908.5	Fuc$_2$Hex$_5$HexNAc$_6$	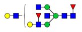
2921.5	Neu5Ac$_1$Hex$_5$HexNAc$_6$	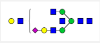
2966.5	Neu5Ac$_2$Fuc$_1$Hex$_5$HexNAc$_4$	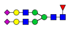

Table S5: ROC curves were generated for free glycans using 89 patients with AD. 86 disease control patients and 87 healthy control patients.

m/z	Composition	AD – Disease Control	AD – Healthy Control	Disease Control – Healthy Control
**273.1**	Hex_1	0.85	0.76	0.45
**314.2**	$HexNAc_1$	0.55	0.39	0.37
**430.2**	$Neu5Ac_1$	0.51	0.55	0.53
**518.3**	$Hex_1HexNAc_1$	0.24	0.15	0.44
**559.3**	$HexNAc_2$	0.41	0.25	0.34
**675.3**	$NeuAc_1HexNAc_1$	0.29	0.13	0.30
**722.4**	$Hex_2HexNAc_1$	0.38	0.17	0.33
**733.4**	$Fuc_1HexNAc_2$	0.27	0.26	0.50
**791.4**	$Neu5Ac_2$	0.46	0.53	0.57
**825.4**	Fuc_2Hex_2	0.39	0.39	0.49
**879.4**	$NeuAc_1Hex_1HexNAc_1$	0.21	0.30	0.59
**926.5**	$Hex_3HexNAc_1$	0.32	0.25	0.46
**967.5**	$Hex_2HexNAc_2$	0.34	0.28	0.50
**1130.6**	$Hex_4HexNAc_1$	0.33	0.33	0.53
**1171.6**	$Hex_3HexNAc_2$	0.39	0.44	0.57
**1212.6**	$Hex_2HexNAc_3$	0.50	0.44	0.44
**1240.6**	$NeuAc_2Hex_1HexNAc_1$	0.39	0.48	0.58
**1334.7**	$Hex_5HexNAc_1$	0.35	0.42	0.58
**1601.8**	$NeuAc_3Hex_1HexNAc_1$	0.46	0.71	0.72

Table S6: Correlations were calculated between the free glycans Hex_1 (m/z 273.1), $HexNAc_1Hex_1Neu5Ac_1$ (m/z 879.4) and CSF parameters using the using the Spearman´s rank correlation coefficient, $p< 0.05$ was considered as statistically significant.

Hex_1							
	MMSE	Age of patients	Aβ40	Aβ42	Aβ-Ratio	pTau	tTau
**AD**	0,232	0,550	0,001	0,147	0,238	0,031	0,024
**HC**	0,792	0,943	0,002	0,031	0,598	0,000	0,015
**DC**	0,001	0,645	0,000	0,000	0,239	0,264	0,888
$HexNAc_1Hex_1Neu5Ac_1$							
**AD**	0,178	0,430	0,822	0,742	0,959	0,577	0,749
**HC**	0,508	0,321	0,000	0,068	0,585	0,004	0,060
**DC**	0,051	0,400	0,009	0,029	0,559	0,961	0,227

Table S7: Mean relative areas and standard deviations of N-glycans isolated from the three patient cohorts as judged by MALDI-TOF-MS. AD: Alzheimer´s disease, DC: disease control and HC: healthy control.

m/z	**Composition**	**AD patients [%]**		**Disease control patients [%]**		**Healthy control patients [%]**	
		**Males**	**Females**	**Males**	**Females**	**Males**	**Females**
**1579.8**	$Hex_5HexNAc_2$	6.13 ± 1.74	6.74 ± 1.69	5.84 ± 1.94	7.36 ± 2.66	7.23 ± 2.23	7.80 ± 2.79
**1661.8**	$Hex_3HexNAc_4$	1.12 ± 0.36	1.00 ± 0.36	1.21 ± 0.43	1.40 ± 0.54	1.10 ± 0.34	1.17 ± 0.32
**1783.9**	$Hex_6HexNAc_2$	0.99 ± 0.19	1.05 ± 0.19	1.12 ± 0.36	1.10 ± 0.29	1.11 ± 0.23	1.13 ± 0.24
**1835.9**	$Fuc_1Hex_3HexNAc_4$	4.65 ± 1.13	4.10 ± 1.16	4.74 ± 2.21	5.46 ± 2.29	4.82 ± 1.69	4.13 ± 1.11
**2040.0**	$Fuc_1Hex_4HexNAc_4$	3.98 ± 0.79	3.38 ± 0.93	3.89 ± 1.65	4.02 ± 1.19	4.22 ± 1.30	3.75 ± 1.07
**2070.0**	$Hex_5HexNAc_4$	0.99 ± 0.21	0.98 ± 0.19	0.97 ± 0.29	0.99 ± 0.29	1.01 ± 0.23	1.02 ± 0.17
**2081.1**	$Fuc_1Hex_3HexNAc_5$	17.38 ± 3.73	17.68 ± 2.52	15.20 ± 5.71	17.96 ± 3.55	18.41 ± 3.74	18.91 ± 4.20
**2111.1**	$Hex_4HexNAc_5$	1.09 ± 0.33	1.05 ± 0.36	1.22 ± 0.43	1.30 ± 0.35	1.14 ± 0.38	1.13 ± 0.26
**2214.1**	$Fuc_2Hex_4HexNAc_4$	1.40 ± 0.30	1.37 ± 0.31	1.14 ± 0.32	1.35 ± 0.32	1.30 ± 0.26	1.26 ± 0.24
**2244.1**	$Fuc_1Hex_5HexNAc_4$	1.73 ± 0.31	1.54 ± 0.29	1.64 ± 0.56	1.54 ± 0.33	1.87 ± 0.45	1.72 ± 0.37
**2285.2**	$Fuc_1Hex_4HexNAc_5$	6.64 ± 1.28	6.54 ± 0.96	5.93 ± 2.17	6.41 ± 1.44	6.31 ± 1.17	6.24 ± 1.04
**2431.2**	$Neu5Ac_1Hex_5HexNAc_4$	2.97 ± 0.85	3.24 ± 1.24	2.90 ± 1.64	2.73 ± 0.94	3.00 ± 1.12	2.74 ± 1.13
**2459.2**	$Fuc_2Hex_4HexNAc_5$	6.34 ± 1.37	7.11 ± 1.46	5.34 ± 2.22	6.19 ± 1.65	7.11 ± 1.44	8.00 ± 1.91
**2489.3**	$Fuc_1Hex_5HexNAc_5$	1.94 ± 0.40	2.11 ± 0.38	1.78 ± 0.60	1.85 ± 0.39	1.87 ± 0.34	1.97 ± 0.42
**2592.3**	$Fuc_3Hex_5HexNAc_4$	1.09 ± 0.29	1.10 ± 0.27	0.95 ± 0.35	0.98 ± 0.36	1.07 ± 0.26	1.03 ± 0.24
**2605.3**	$Neu5Ac_1Fuc_1Hex_5HexNAc_4$	3.23 ± 0.60	3.04 ± 0.44	2.73 ± 0.91	2.55 ± 0.63	2.91 ± 0.57	2.68 ± 0.55
**2646.3**	$Neu5Ac_1Fuc_1Hex_4HexNAc_5$	2.17 ± 0.51	2.08 ± 0.49	1.82 ± 0.70	1.75 ± 0.60	1.75 ± 0.48	1.63 ± 0.42
**2792.4**	$Neu5Ac_2Hex_5HexNAc_4$	8.58 ± 3.30	8.87 ± 3.47	8.16 ± 4.82	7.62 ± 4.13	7.66 ± 3.60	7.07 ± 4.21
**2820.4**	$Neu5Ac_1Fuc_2Hex_4HexNAc_5$	1.03 ± 0.37	1.02 ± 0.28	0.91 ± 0.37	0.89 ± 0.38	0.92 ± 0.32	0.93 ± 0.35
**2837.4**	$Fuc_3Hex_5HexNAc_5$	1.10 ± 0.35	1.23 ± 0.34	1.03 ± 0.46	1.01 ± 0.42	1.19 ± 0.40	1.28 ± 0.34
**2850.4**	$Neu5Ac_1Fuc_1Hex_5HexNAc_5$	2.12 ± 0.57	2.23 ± 0.59	1.90 ± 0.88	1.85 ± 0.71	1.80 ± 0.55	1.75 ± 0.60
**2966.5**	$Neu5Ac_2Fuc_1Hex_5HexNAc_4$	2.64 ± 0.85	2.35 ± 0.53	2.25 ± 1.11	2.07 ± 1.09	2.03 ± 0.71	1.87 ± 0.92
**other**		20.24	19.70	25.56	21.05	19.70	20.23

Table S8: Mean relative areas and standard deviations of O-glycans isolated from the three patient cohorts as judged by MALDI-TOF-MS. AD: Alzheimer´s disease, DC: disease control and HC: healthy control. As m/z 273.1 and 1124.6 were gender-dependent (Supplementary Material Table S3), mean relative areas and standard deviations were calculated separately.

m/z	**Composition**	**AD patients [%]**		**Disease control patients [%]**		**Healthy control patients [%]**	
		**Males**	**Females**	**Males**	**Females**	**Males**	**Females**
**273.1**	Hex$_1$	22.9 ± 11.1	16.3 ± 10.5	15.3 ± 5.8	15.0 ± 8.2	12.7 ± 4.9	13.2 ± 4.4
**1124.6**	Neu5Ac$_1$Hex$_1$HexNAc$_2$	0.8 ± 0.6	0.7 ± 0.4	0.8 ± 0.5	0.6 ± 0.3	1.0 ± 0.7	0.9 ± 0.4
**314.2**	HexNAc$_1$	44.2 ± 11.2		50.0 ± 10.6		45.0 ± 12.1	
**518.3**	Hex$_1$HexNAc$_1$	8.2 ± 2.0		10.1 ± 4.3		8.5 ± 2.5	
**559.3**	HexNAc$_2$	5.4 ± 1.4		4.3 ± 1.3		4.9 ± 1.1	
**675.3**	Neu5Ac$_1$HexNAc$_1$	3.0 ± 1.3		2.8 ± 1.6		3.6 ± 1.3	
**722.4**	Hex$_2$HexNAc$_1$	3.6 ± 1.1		3.1 ± 1.1		4.2 ± 1.2	
**763.8**	Hex$_1$HexNAc$_2$	3.0 ± 1.1		2.6 ± 1.1		3.5 ± 1.2	
**804.4**	HexNAc$_3$	2.3 ± 1.5		2.4 ± 1.5		3.3 ± 1.8	
**825.4**	Fuc$_2$Hex$_2$	2.3 ± 1.0		2.1 ± 1.0		2.9 ± 1.2	
**879.4**	Neu5Ac$_1$Hex$_1$HexNAc$_1$	3.0 ± 1.2		2.7 ± 1.5		4.3 ± 2.1	
**967.5**	Hex$_2$HexNAc$_2$	2.0 ± 1.0		1.9 ± 1.1		2.5 ± 1.3	
**1240.6**	Neu5Ac$_2$Hex$_1$HexNAc$_1$	0.6 ± 0.5		0.6 ± 0.6		1.2 ± 1.0	

Table S9: Glycan types and Glycosylation features used to generate the mean relative intensities presented in Figure 3. Bold: sialylated glycans.

Glycan type	Subcategory	*Relative intensities of MS signals at m/z*
Free	N: N-glycans	733.4, 926.5, 1130.6, 1171.6, 1334.7
	O: O-glycans	825.4, 967.5, 1212.6, **1240.6**, **1601.8**
	Fragments (N-, O-glycans)	273.1, 314.2, 518.3, 559.3, **675.3**, 722.4, **879.4**
	Free Sialic Acids	**430.2**, **791.4**
N-glycans	high-mannose	1579.8, 1783.9
	biantennary	1661.8, 1835.9, 2040.0, 2070.0, 2214.1, 2244.1, **2431.2**, 2592.3, **2605.3**, **2792.4**, **2966.5**
	bisected biantennary	2081.1, 2111.1, 2285.2, 2459.2, 2489.3, **2646.3, 2820.4,** 2837.4, 2850.4
O-glycans		273.1, 314.2, 518.3, 559.3, **675.3,** 722.4, 763.4, 804.4, 825.4, **879.4**, 967.5, **1124.6, 1240.6**

Table S10. Correlations were calculated between N-glycans groups by features and t-Tau using the Spearman´s rank correlation coefficient, p<0.05 and p<0.01 were considered as statistically significant and very significant.

**Group**	**Spearman**	**Spearman**
	**Males**	**Females**
**high-mannose**	0.018	0.616
**asialylated biantennary**	0.183	0.106
**sialylated biantennary**	0.889	0.163
**asialylated bisected**	0.041	0.018
**sialylated bisected**	0.000	0.000
